# Supplementary material for: One-Pot NBS-Promoted Synthesis of Imidazoles and Thiazoles from Ethylarenes in Water
Source: Molecules. 2019 Mar 4;24(5):893. doi: 10.3390/molecules24050893 (PMC6429224; doi:10.3390/molecules24050893)

Supporting Information

One-Pot NBS-Promoted Synthesis of Imidazoles and Thiazoles from Ethylarenes in Water

Liang Chen [1,2], Huajian Zhu [1,2], Jiang Wang [2,*] and Hong Liu [1,2,*]

[1] School of Pharmacy, China Pharmaceutical University, Jiangsu, Nanjing 210009, China; zhulinfengcl@163.com (L.C.); cpuzhj@163.com (H.Z.)

[2] State Key Laboratory of Drug Research and CAS Key Laboratory of Receptor Research, Shanghai Institute of Materia Medica, Chinese Academy of Sciences, Shanghai, Shanghai 201203, China

* Correspondence: jwang@simm.ac.cn (J.W.); hliu@simm.ac.cn (H.L.); Tel.: +86-50807042 (H.L.)

Contents

(A) Optimization of reaction conditions.. S2

(B) Copies of the ^{1}H-NMR and ^{13}C-NMR spectra of all compounds....................................... S4

(A) Optimization of reaction conditions.

Table S1. Optimization of equivalents of oxidant[a]

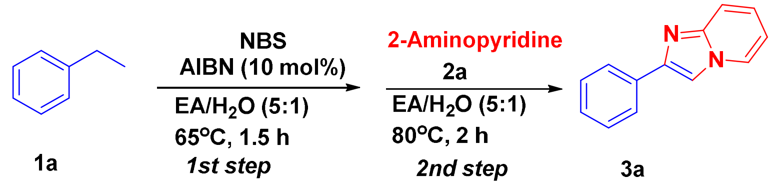

First step			Second step			
Entry.	Oxidant (equiv.)	Solvent	Solvent	Temp. (°C)	Base (equiv.)	Yield[b] (%)
1	NBS (3.5)	EA/H_2O (5:1)	EA/H_2O (5:1)	80	none	38
2	NBS (2.5)	EA/H_2O (5:1)	EA/H_2O (5:1)	80	none	27
3	NBS (4.5)	EA/H_2O (5:1)	EA/H_2O (5:1)	80	none	36

[a] Reactions were run with ethylbenzene **1a** (1 mmol) of with NBS in the presence of AIBN (10 mol%) in EA:H_2O (5:1, 6 mL) at 65 °C for 1.5 h, followed by reaction with 2-aminopyridine **2a** (1.2 mmol) at 80°C for 2 h. [b]Isolated yields.

Table S2. Optimization of solvent and temperature of first step[a]

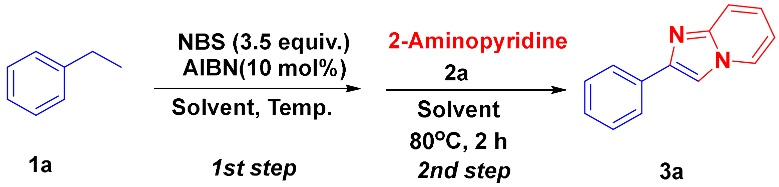

First step				Second step			
Entry.	Oxidant	Solvent	Temp. (°C)	Solvent	Temp. (°C)	Base (equiv.)	Yield[b] (%)
1	NBS	EA/H_2O (5:1)	65	EA/H_2O (5:1)	80	none	38
2	NBS	EA/H_2O (10:1)	65	EA/H_2O (10:1)	80	none	36
3	NBS	EA/H_2O (2:1)	65	EA/H_2O (2:1)	80	none	23
4	NBS	EA/H_2O (5:1)	60	EA/H_2O (5:1)	80	none	35
5	NBS	EA/H_2O (5:1)	70	EA/H_2O (5:1)	80	none	28

(B) Copies of the ^{1}H-NMR and ^{13}C-NMR spectra of all compounds

2-Bromo-1-phenylethan-1-one (1aa)

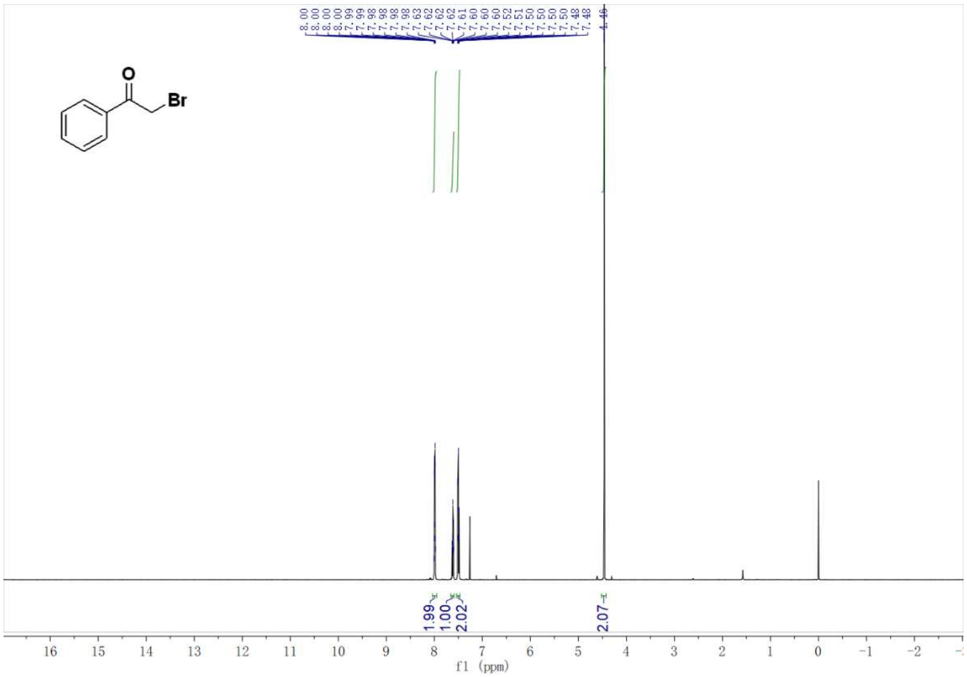

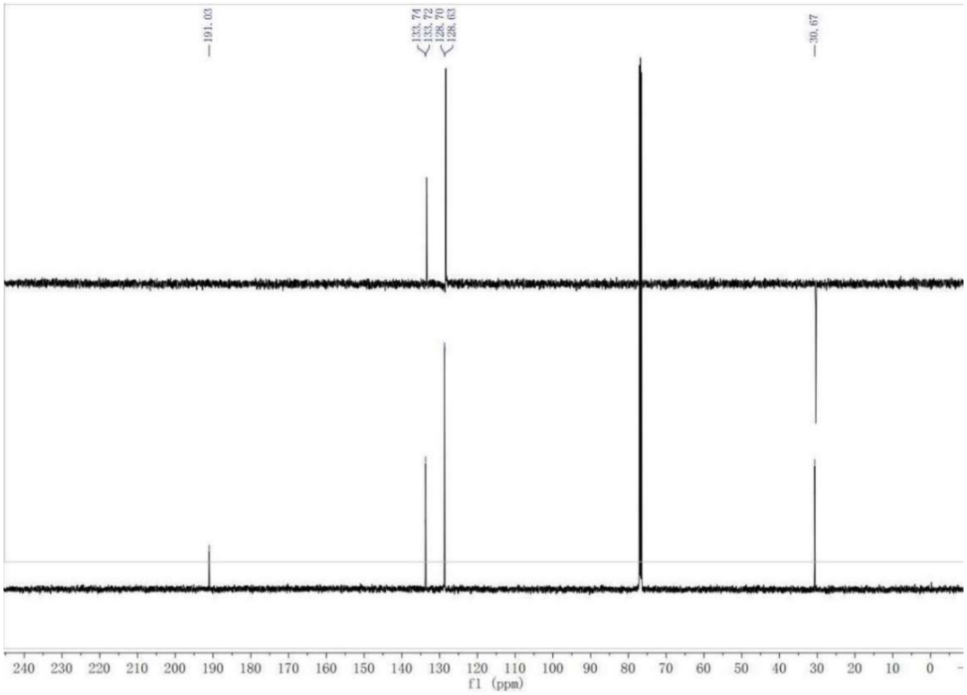

2-Phenylimidazo[1,2-a]pyridine (3a)

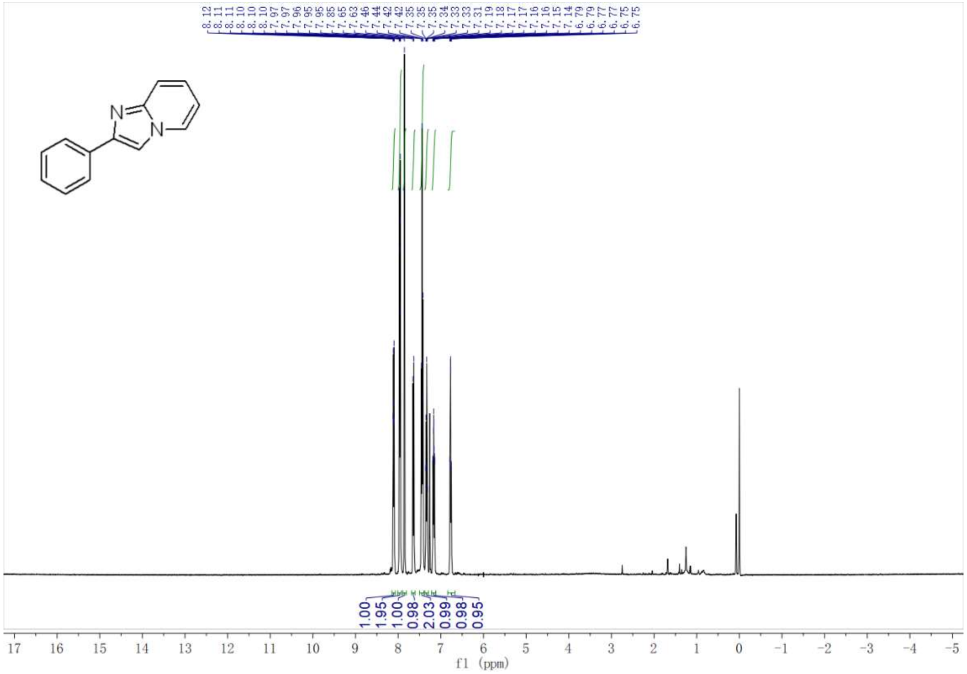

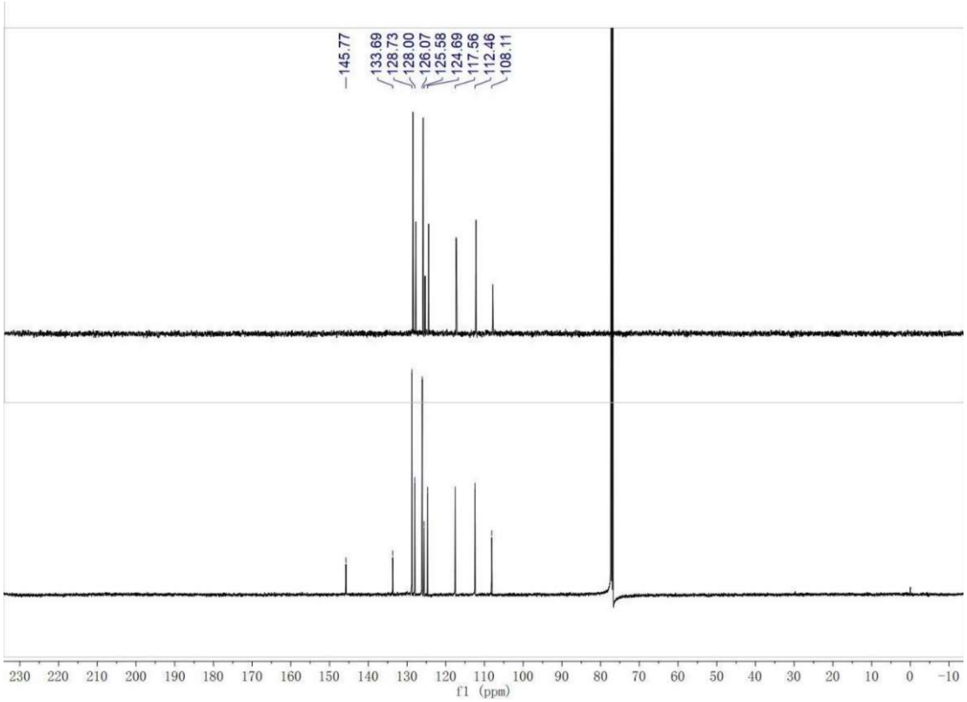

2-(4-Fluorophenyl)imidazo[1,2-a]pyridine (3b)

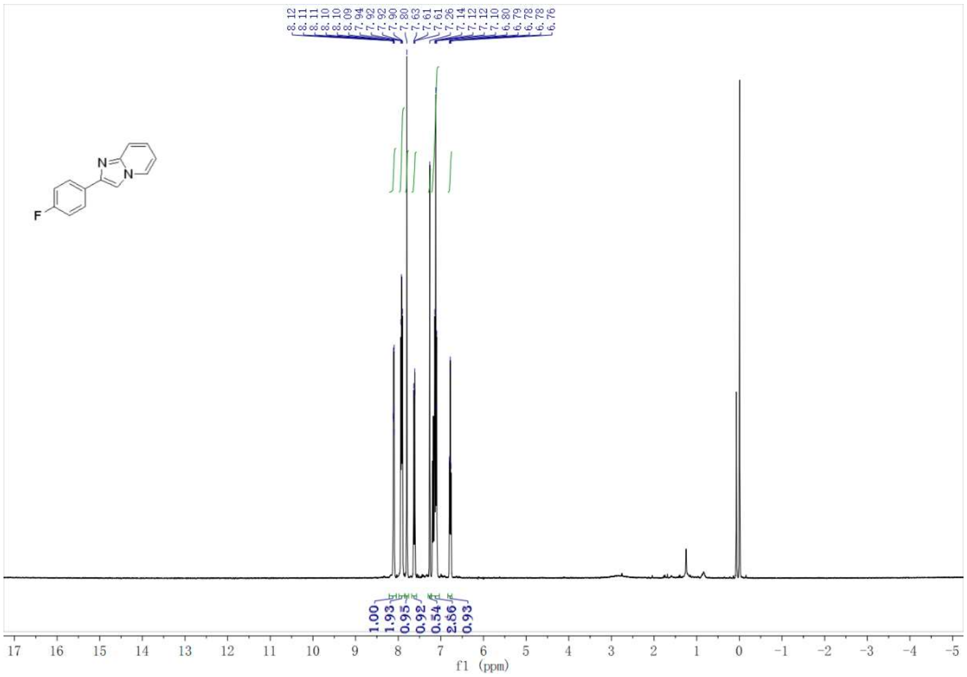

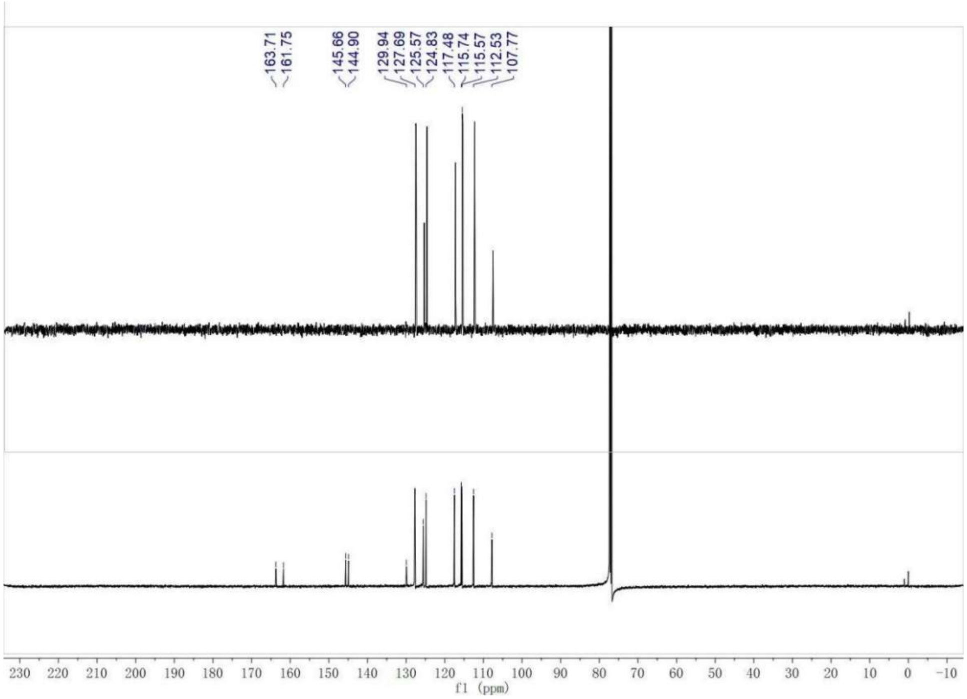

2-(4-Bromophenyl)imidazo[1,2-a]pyridine (3c)

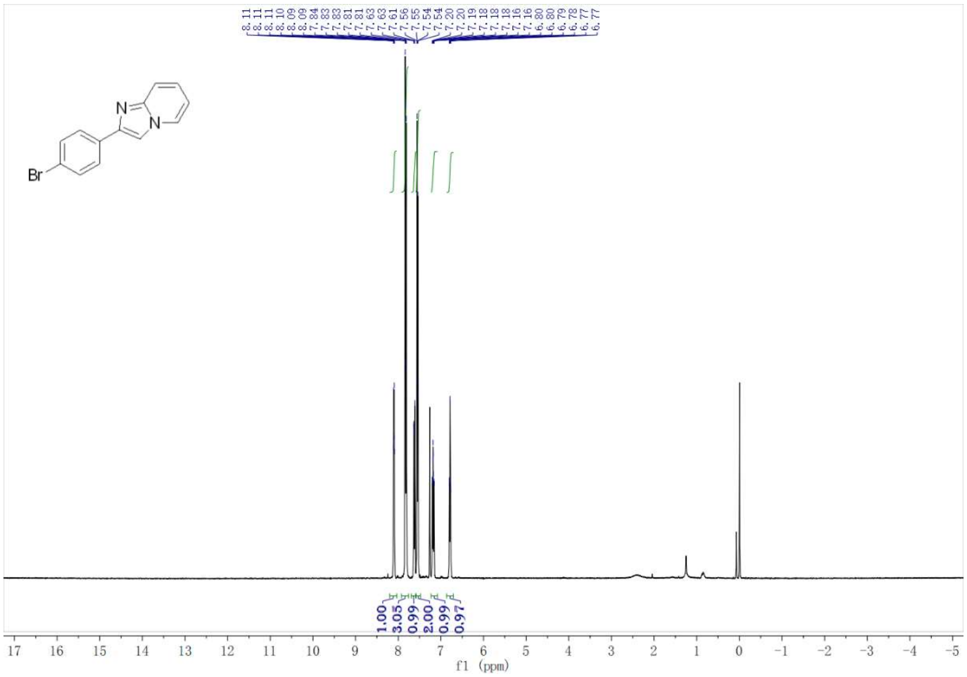

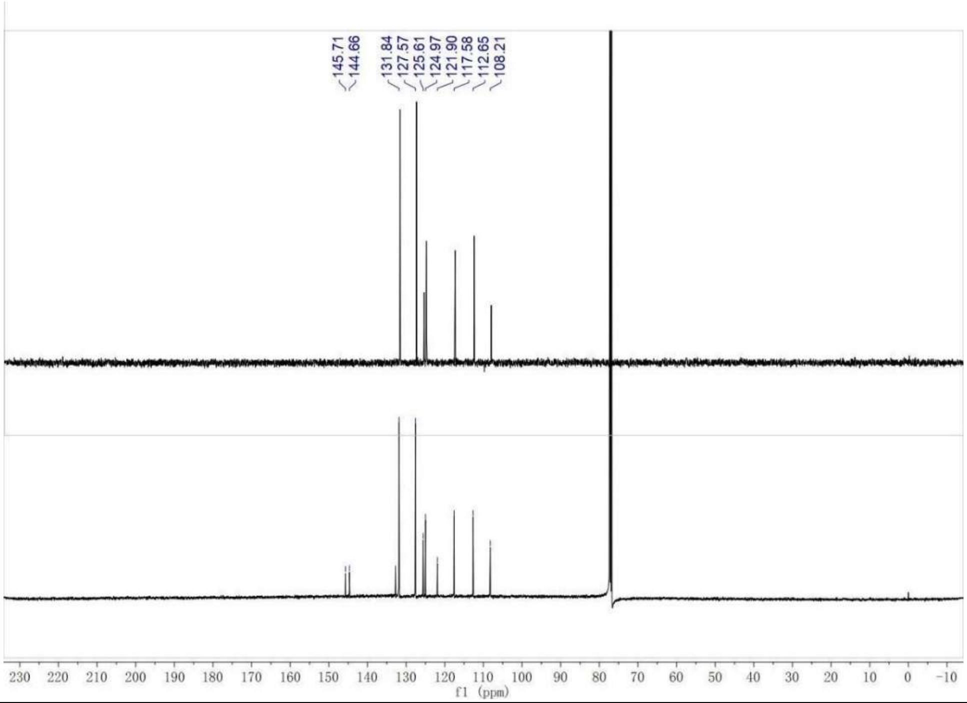

2-(4-Chlorophenyl)imidazo[1,2-a]pyridine (3d)

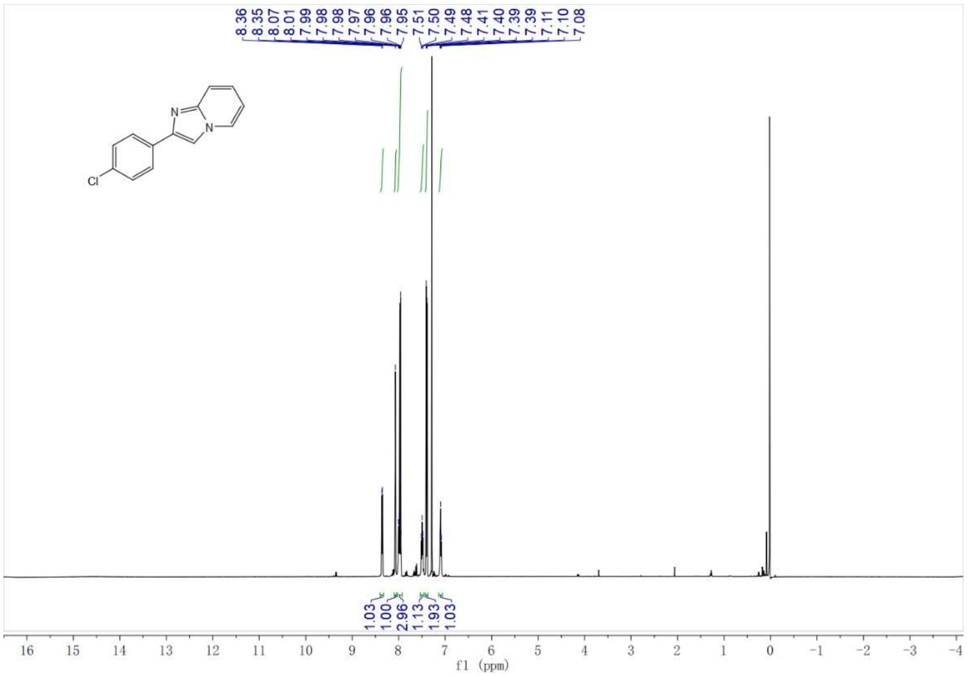

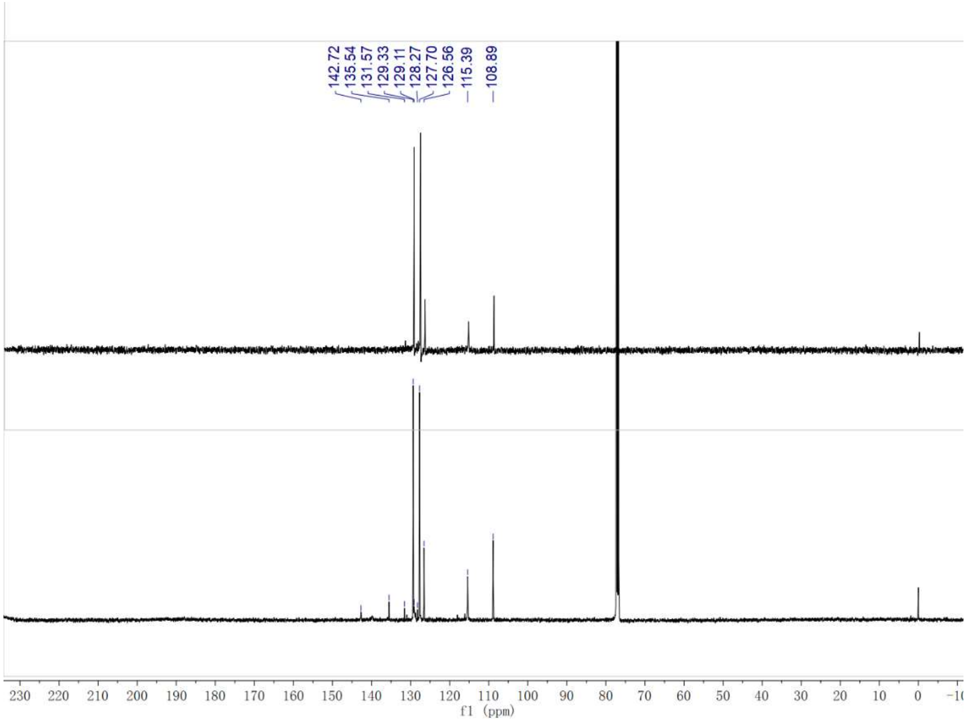

2-(3,5-Difluorophenyl)imidazo[1,2-a]pyridine (3e)

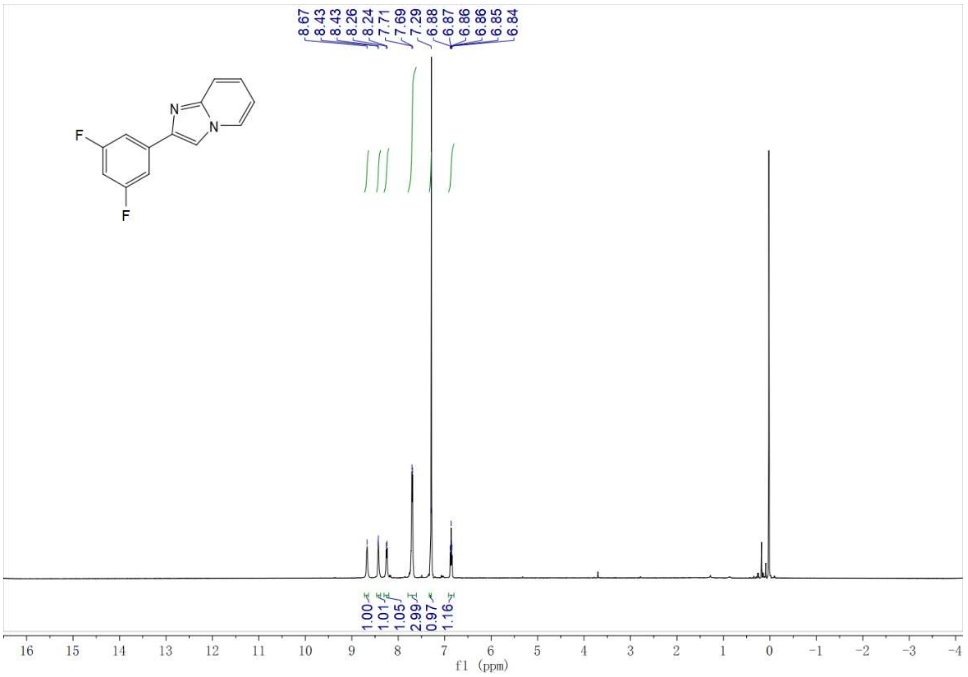

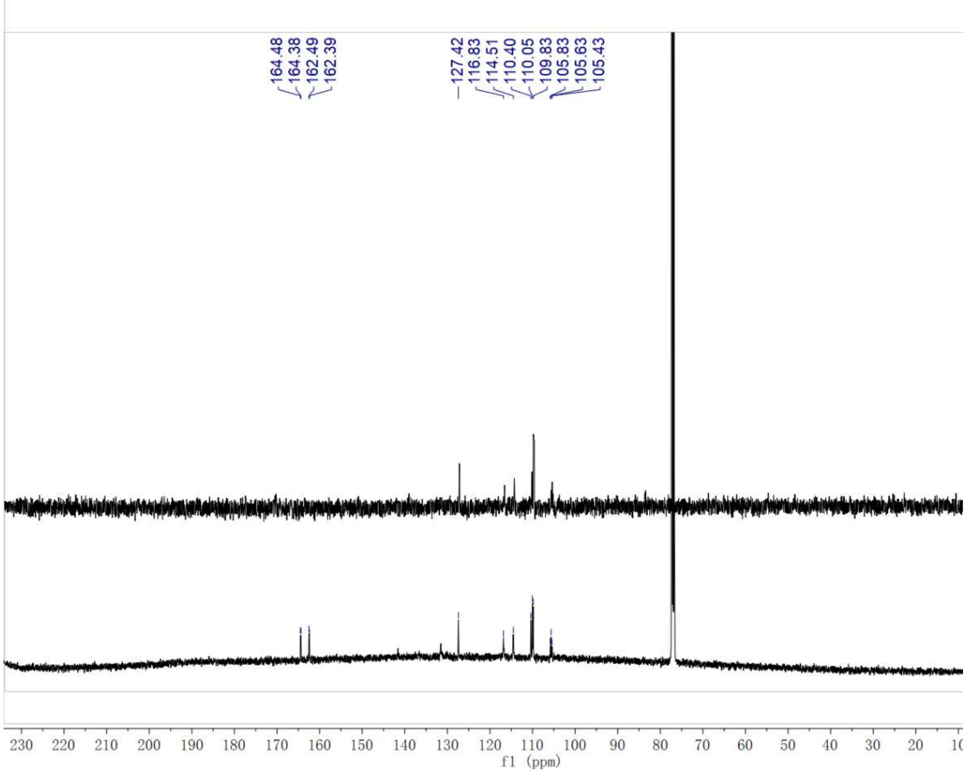

4-(Imidazo[1,2-a]pyridin-2-yl)benzonitrile (3f)

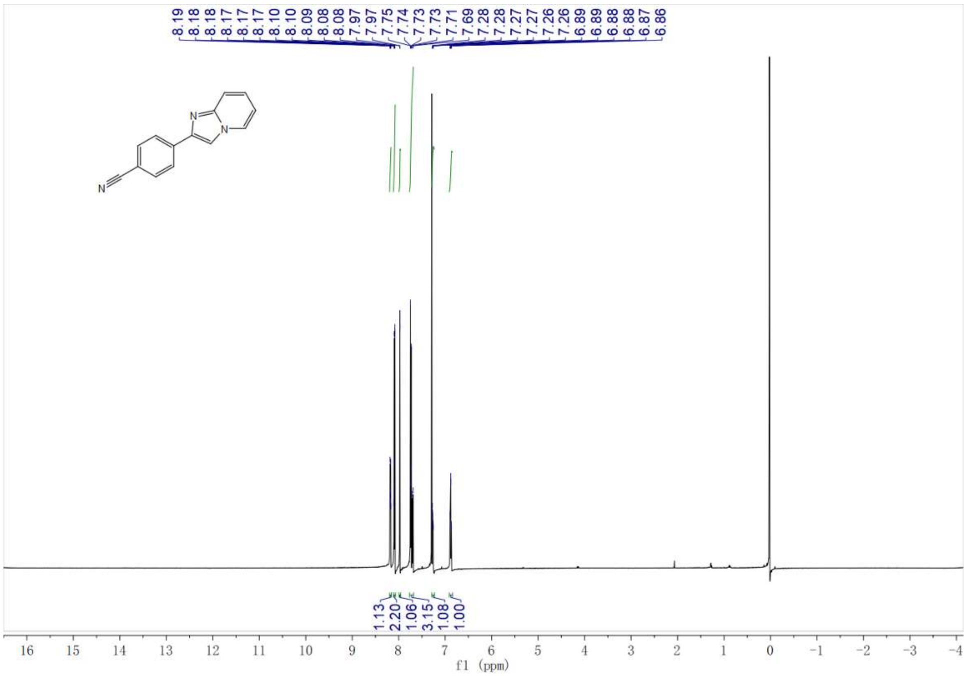

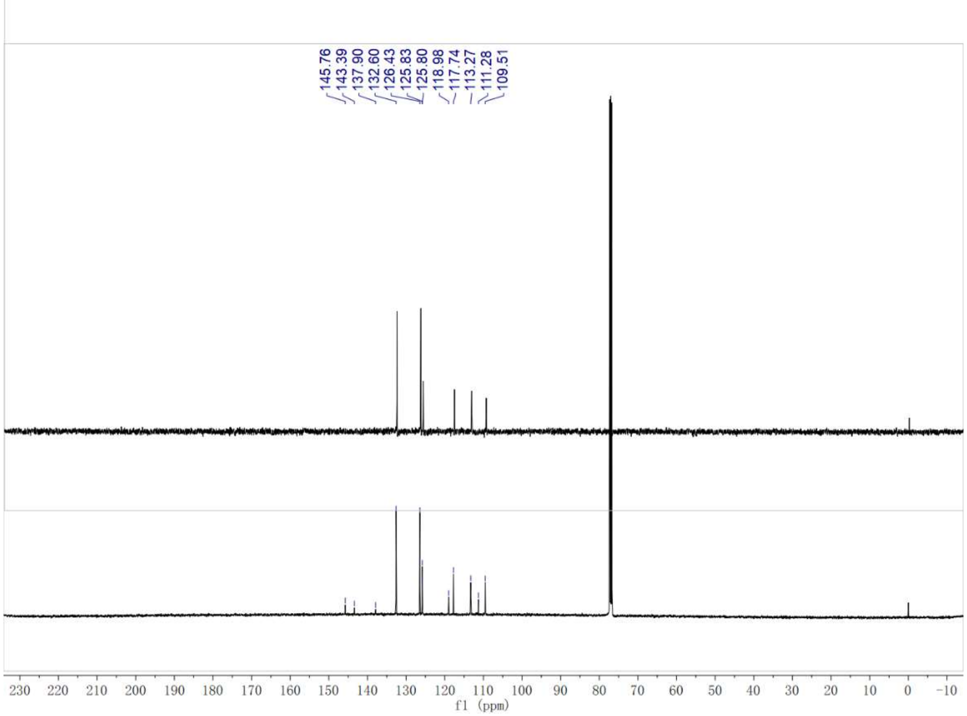

2-(4-(Trifluoromethyl)phenyl)imidazo[1,2-a]pyridine (3g)

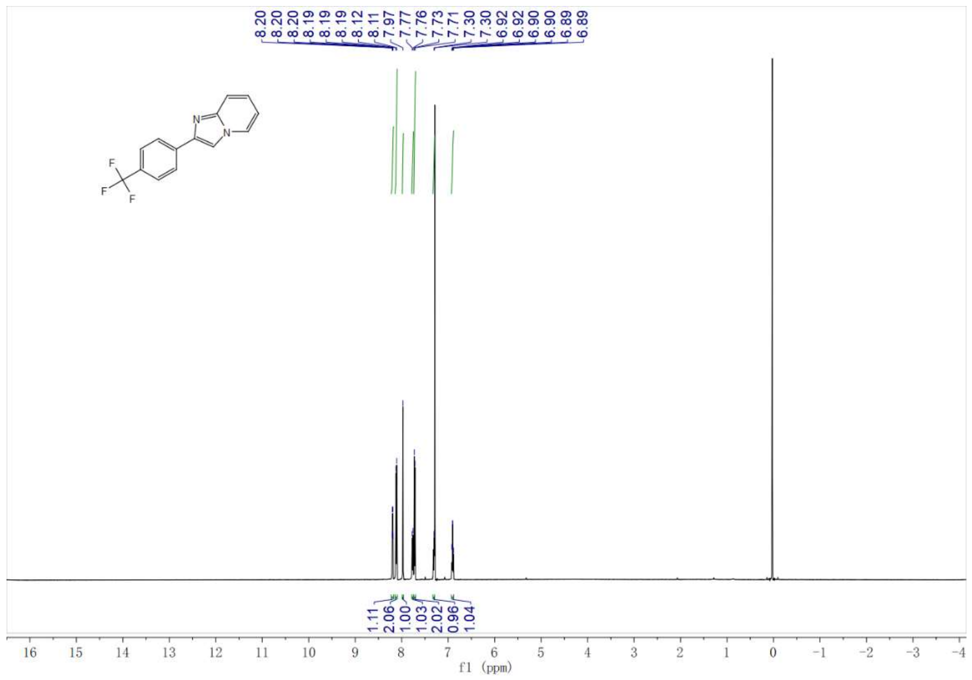

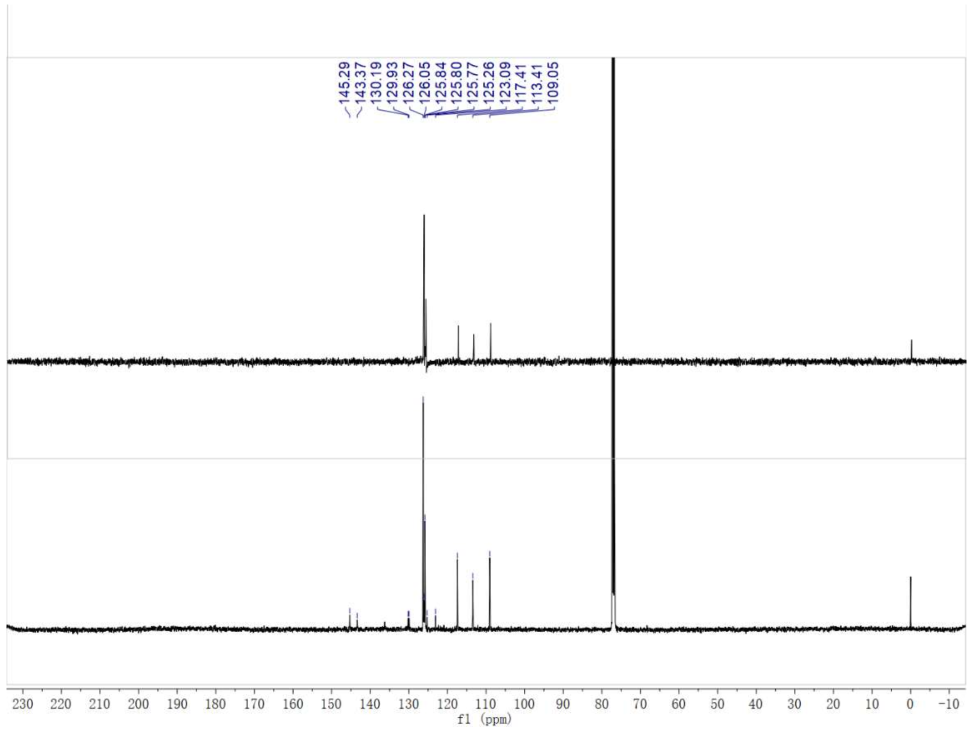

2-(4-(Methylsulfonyl)phenyl)imidazo[1,2-a]pyridine (3h)

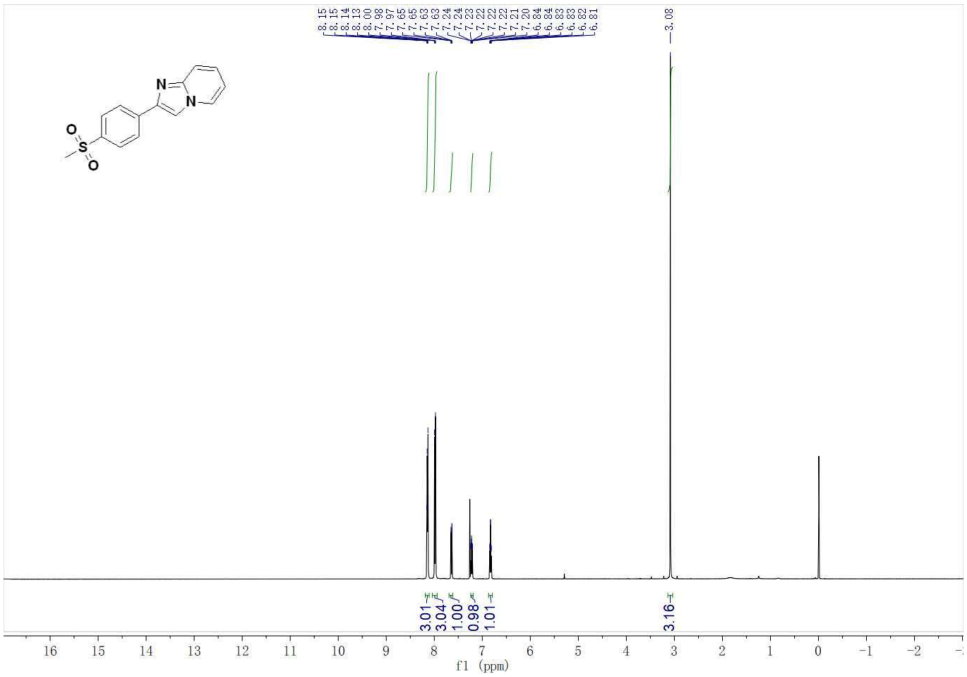

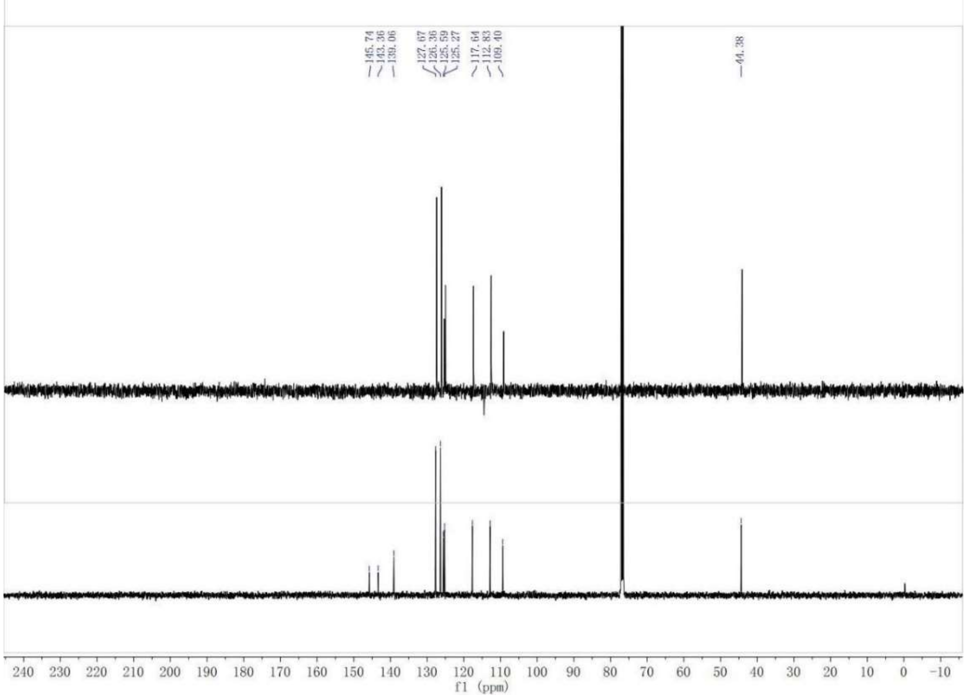

2-(*p*-Tolyl)imidazo[1,2-a]pyridine (3i)

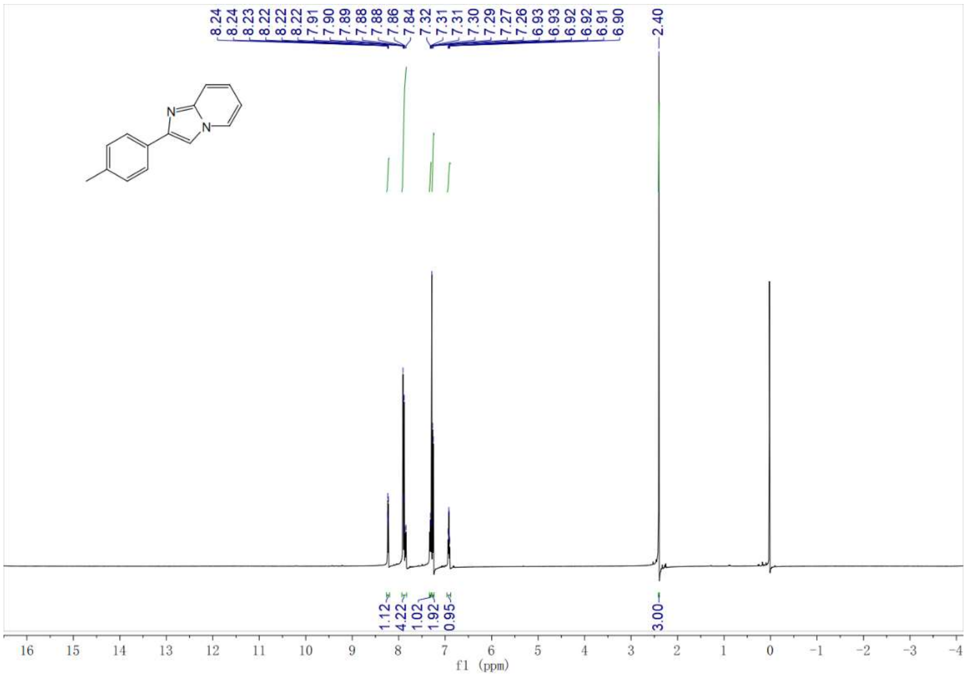

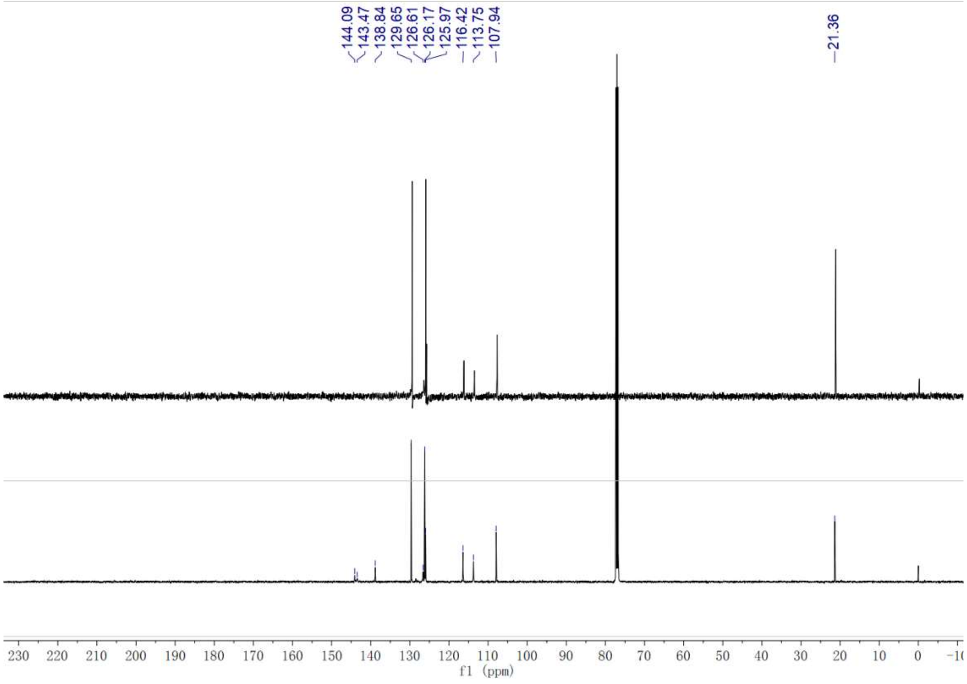

2-(4-Methoxyphenyl)imidazo[1,2-a]pyridine (3j)

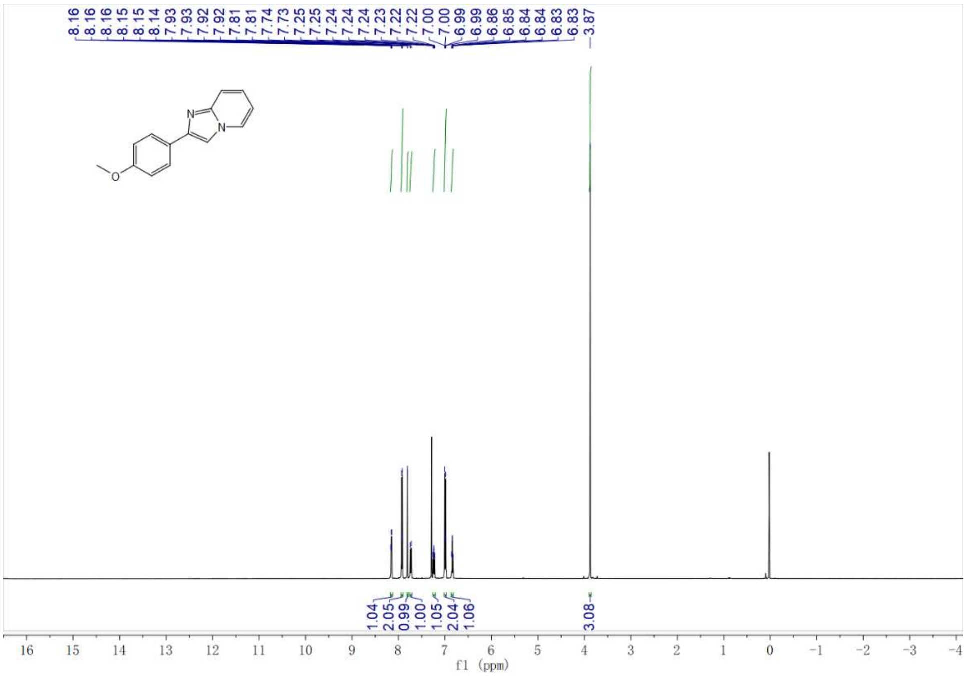

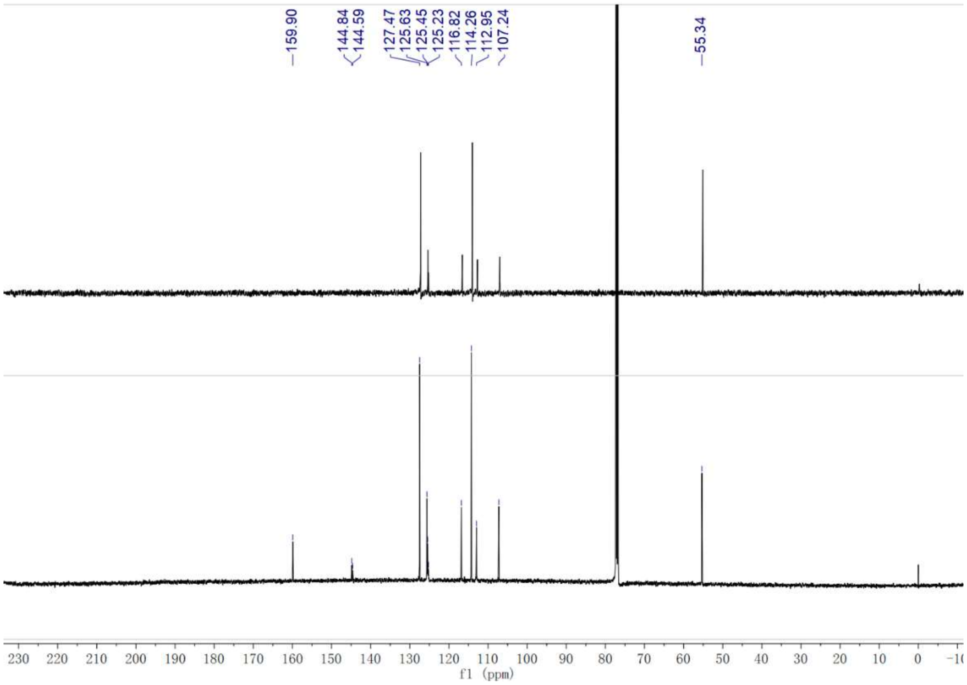

2-(Naphthalen-1-yl)imidazo[1,2-a]pyridine (3k)

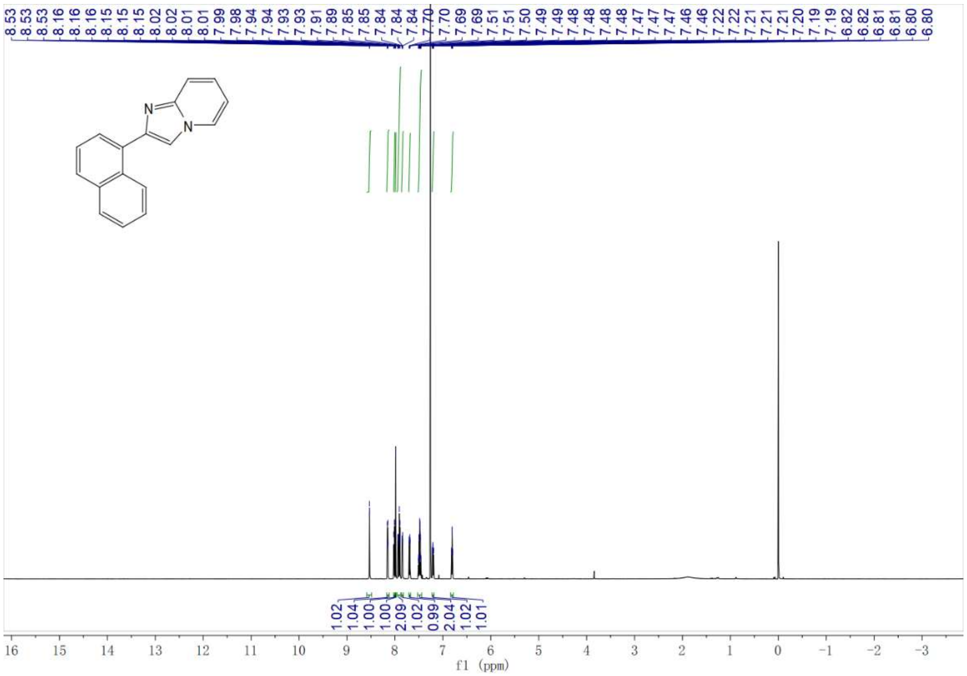

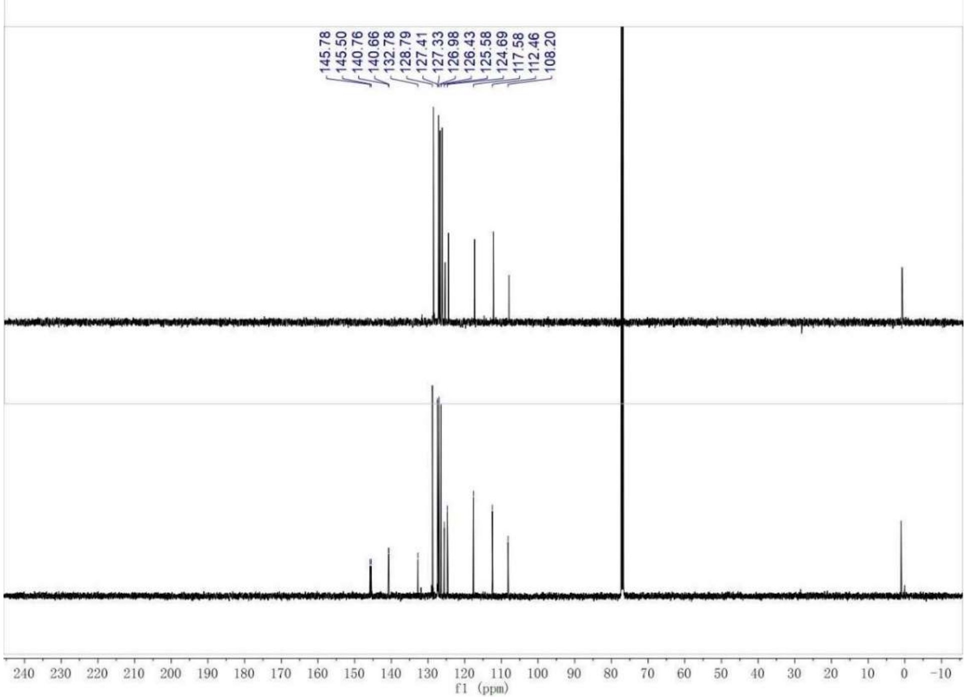

8-Methyl-2-phenylimidazo[1,2-a]pyridine (3l)

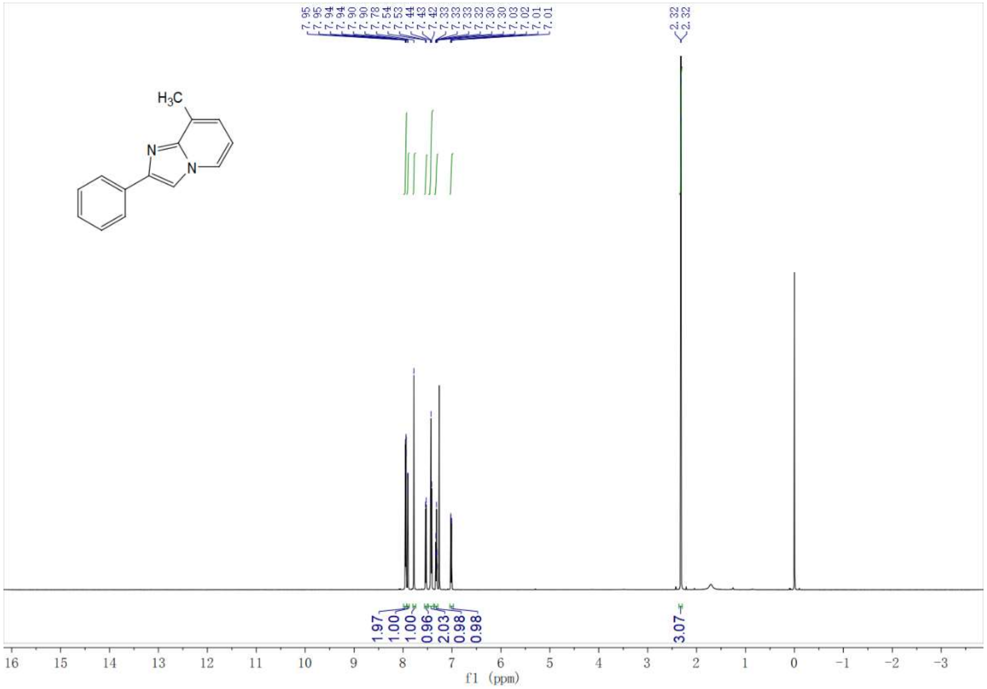

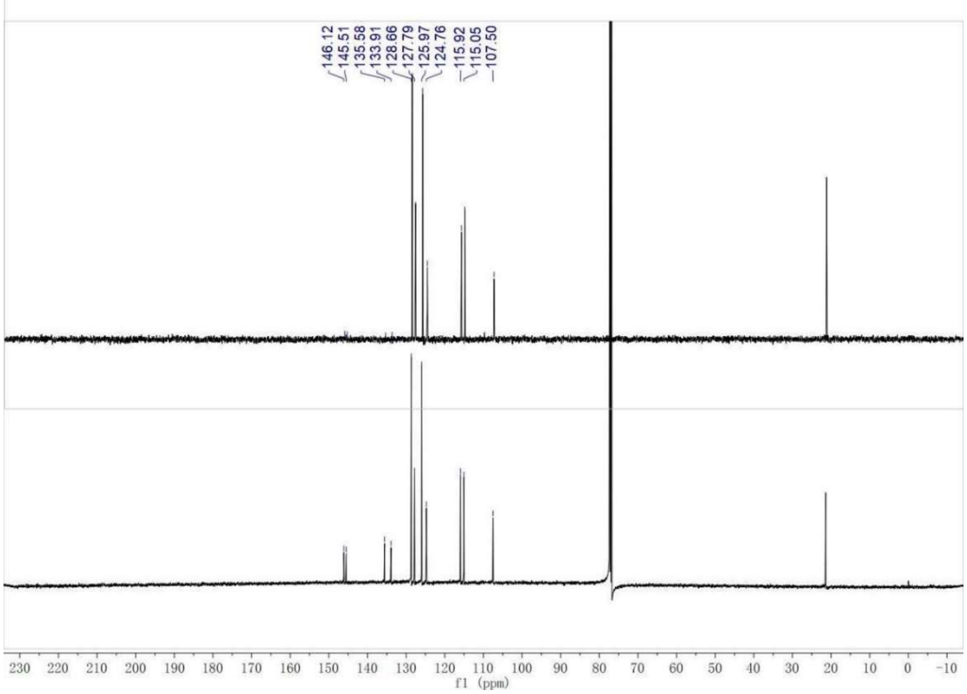

7-Methyl-2-phenylimidazo[1,2-a]pyridine (3m)

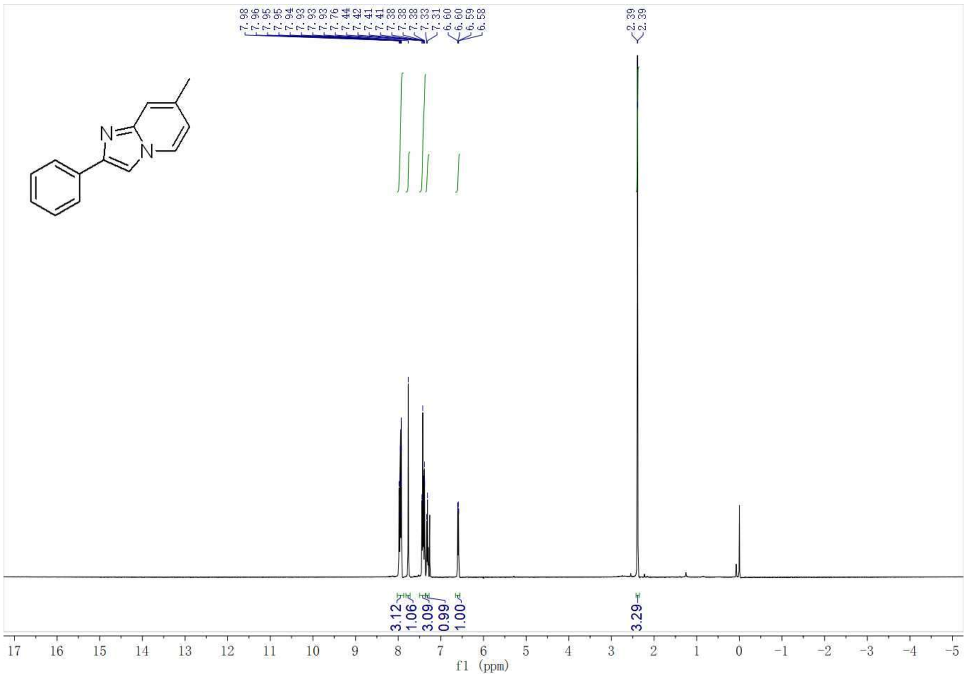

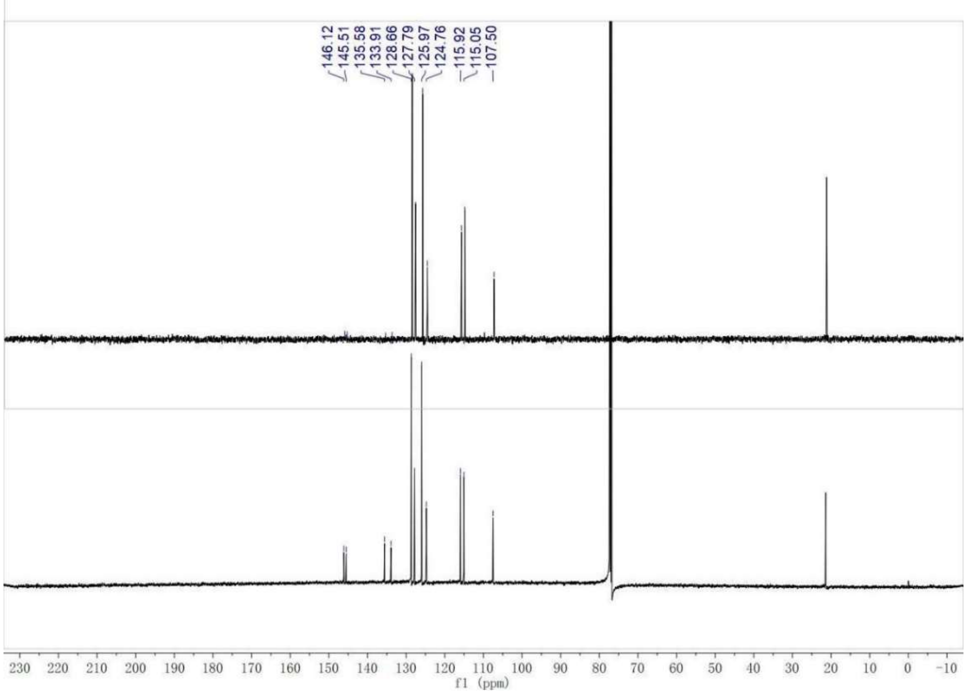

6-Methyl-2-phenylimidazo[1,2-a]pyridine (3n)

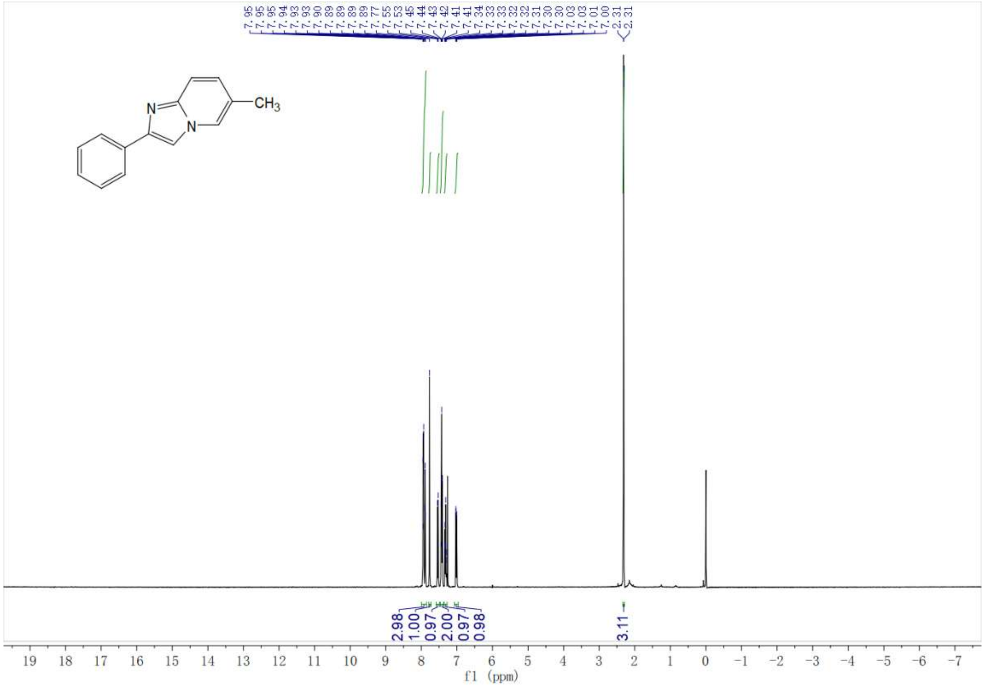

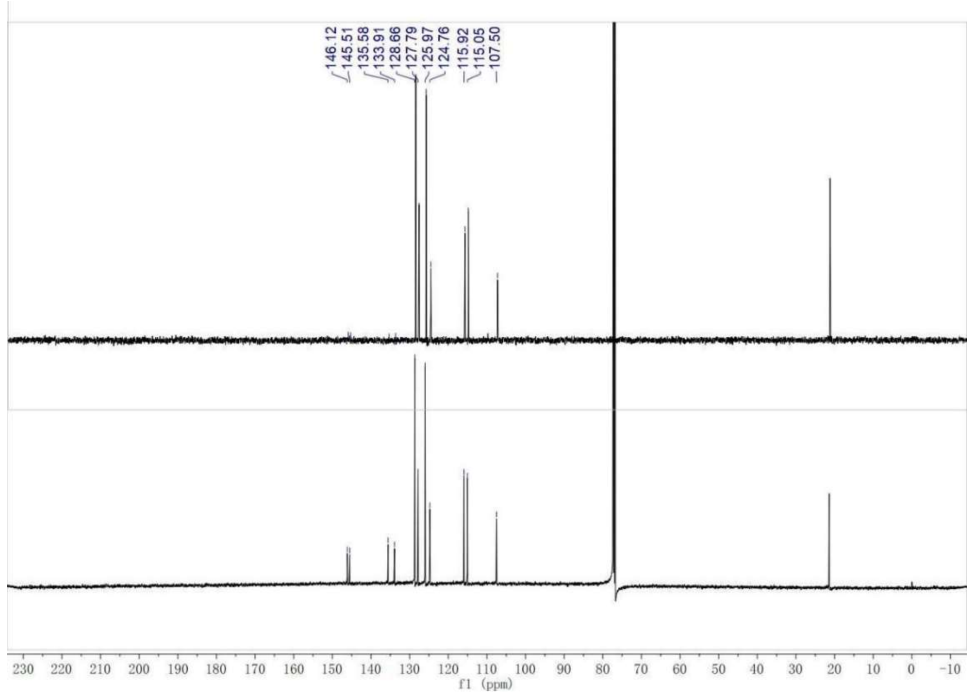

6-Methoxy-2-phenylimidazo[1,2-a]pyridine (3o)

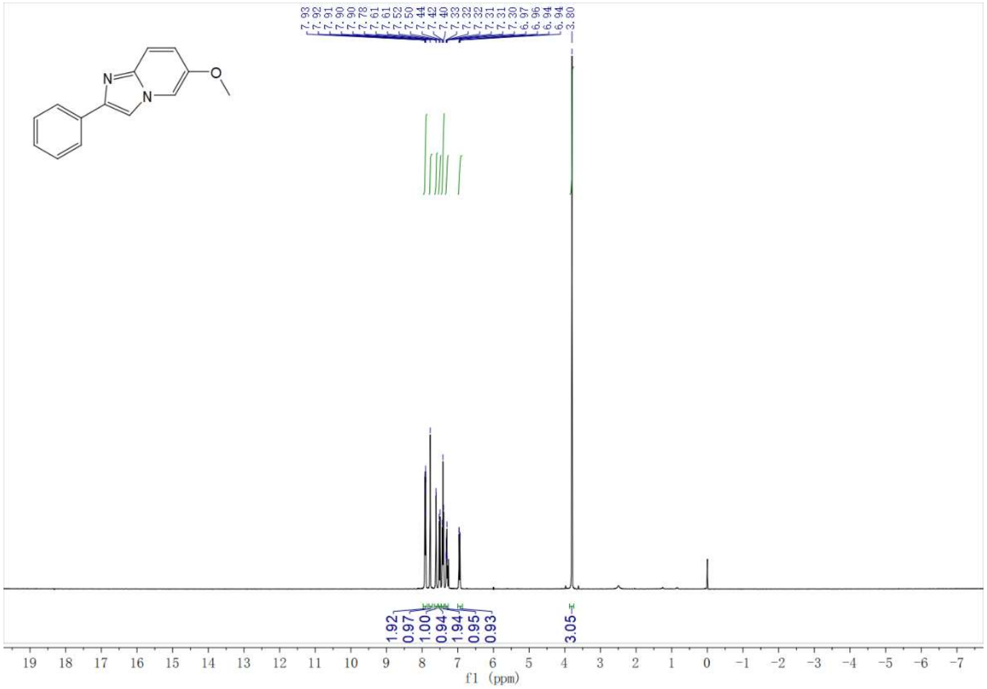

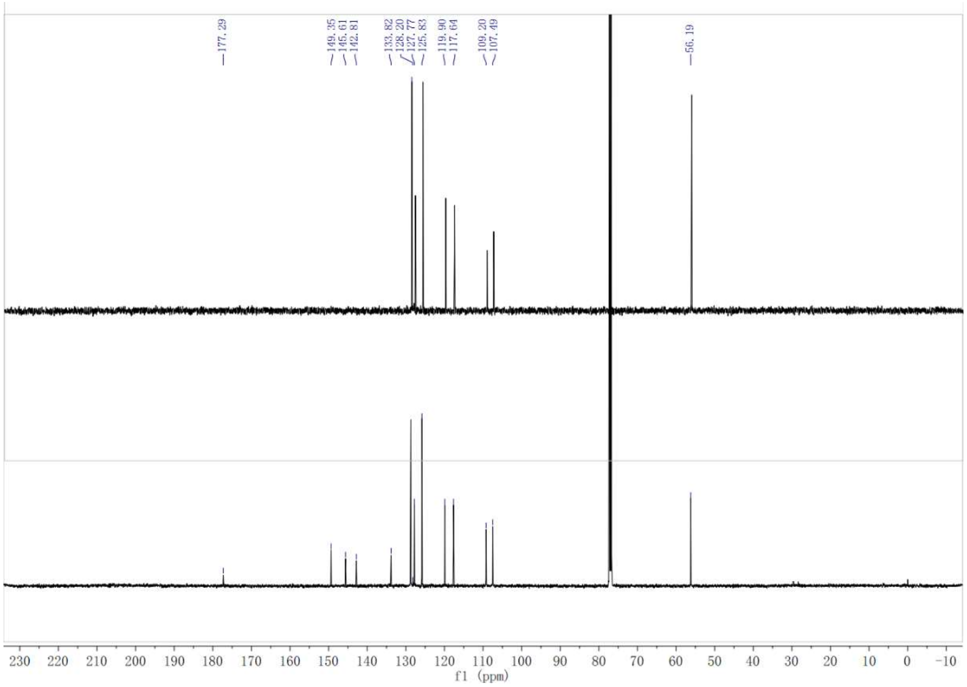

5-Methoxy-2-phenylimidazo[1,2-a]pyridine (3p)

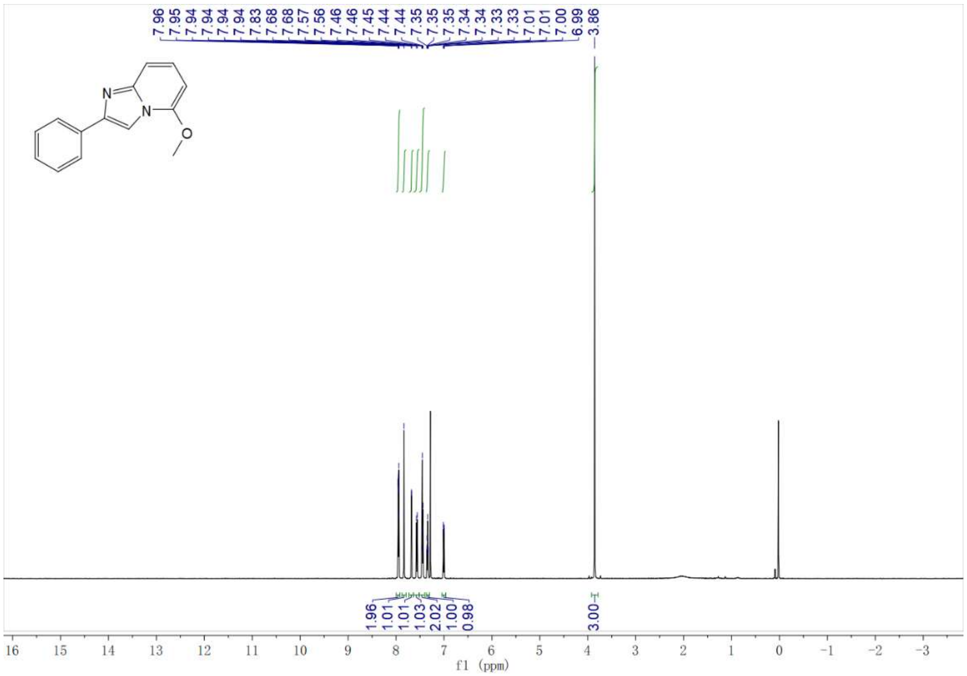

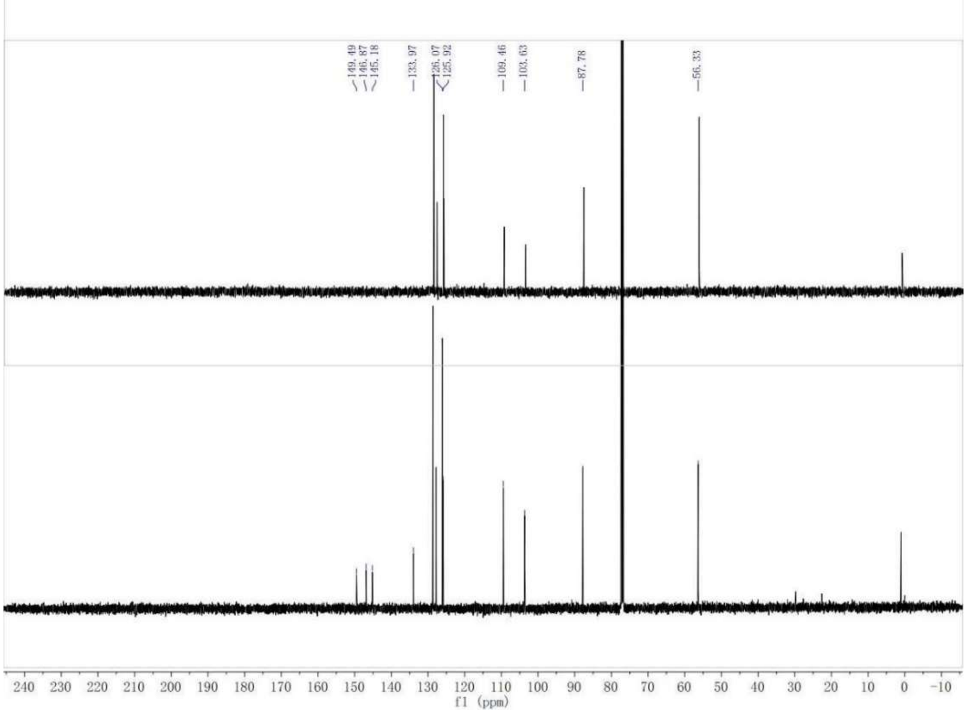

6-Fluoro-2-phenylimidazo[1,2-a]pyridine (3q)

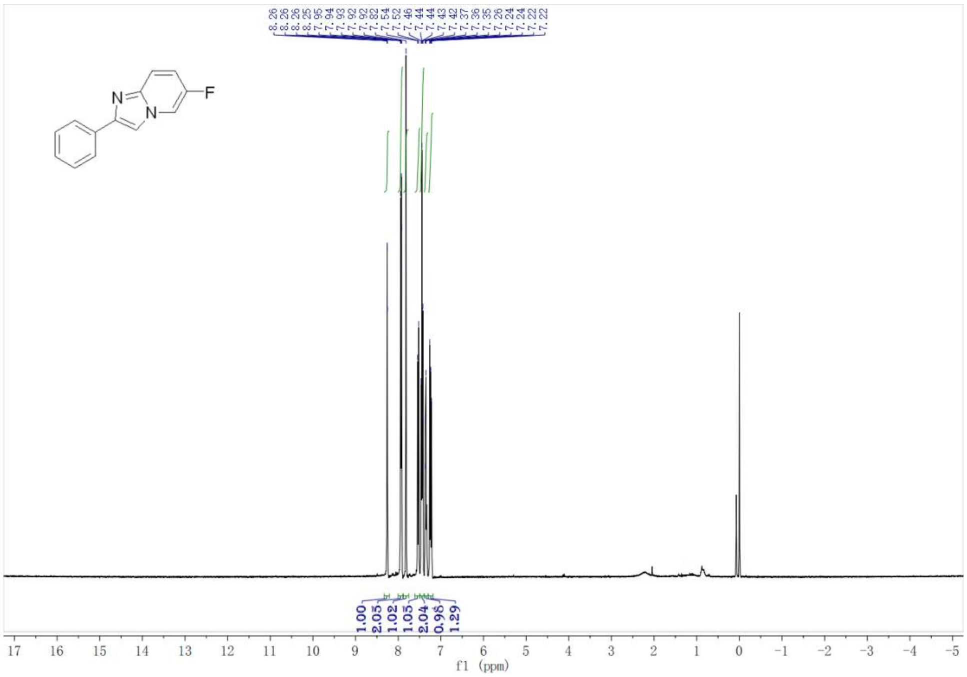

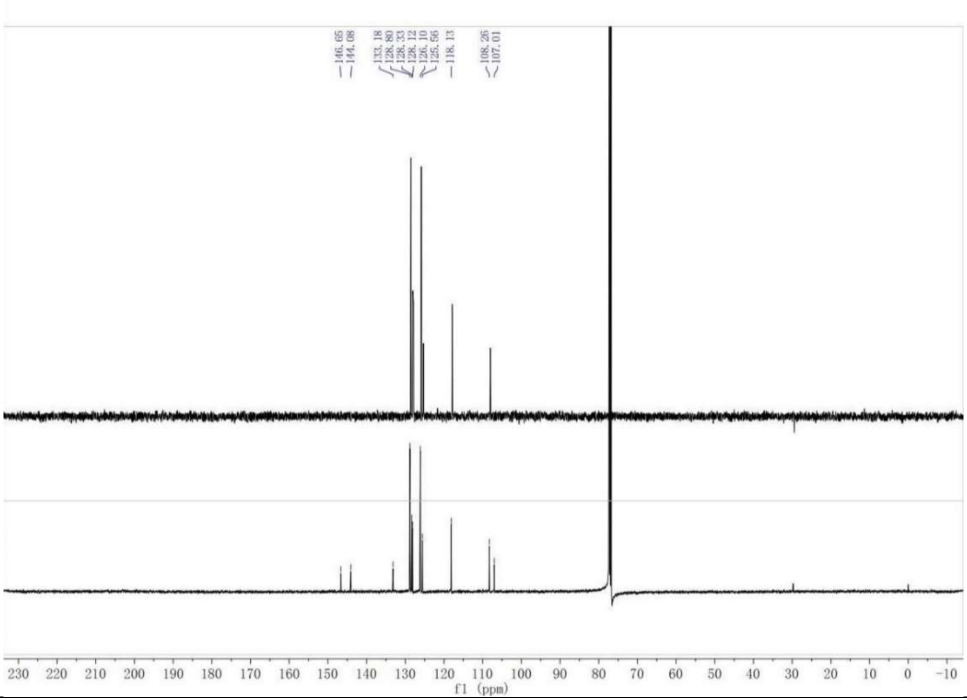

6-Chloro-2-phenylimidazo[1,2-a]pyridine (3r)

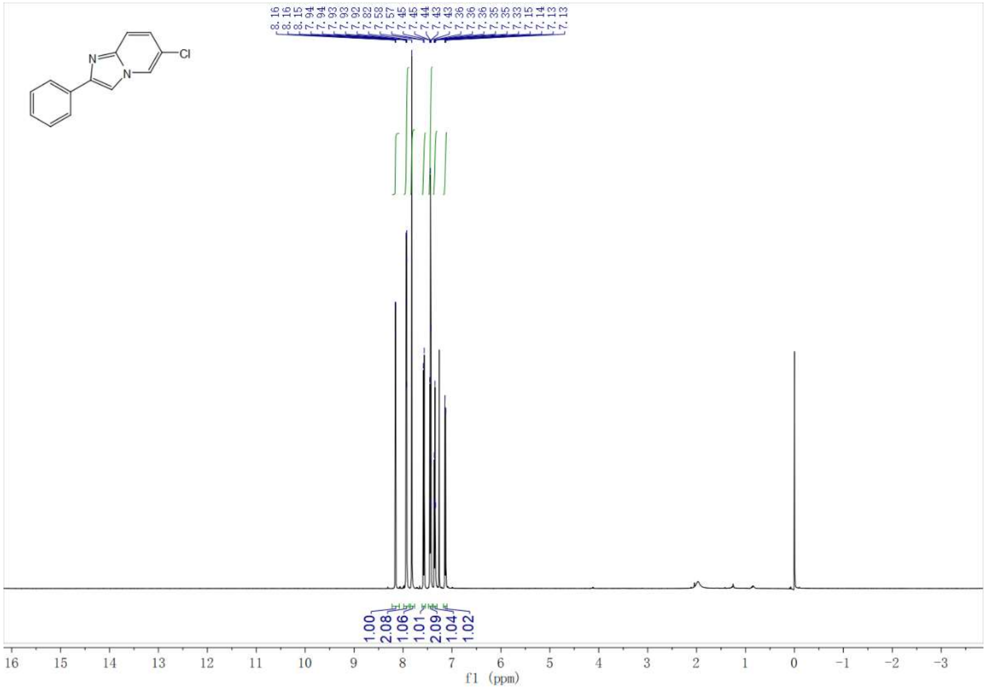

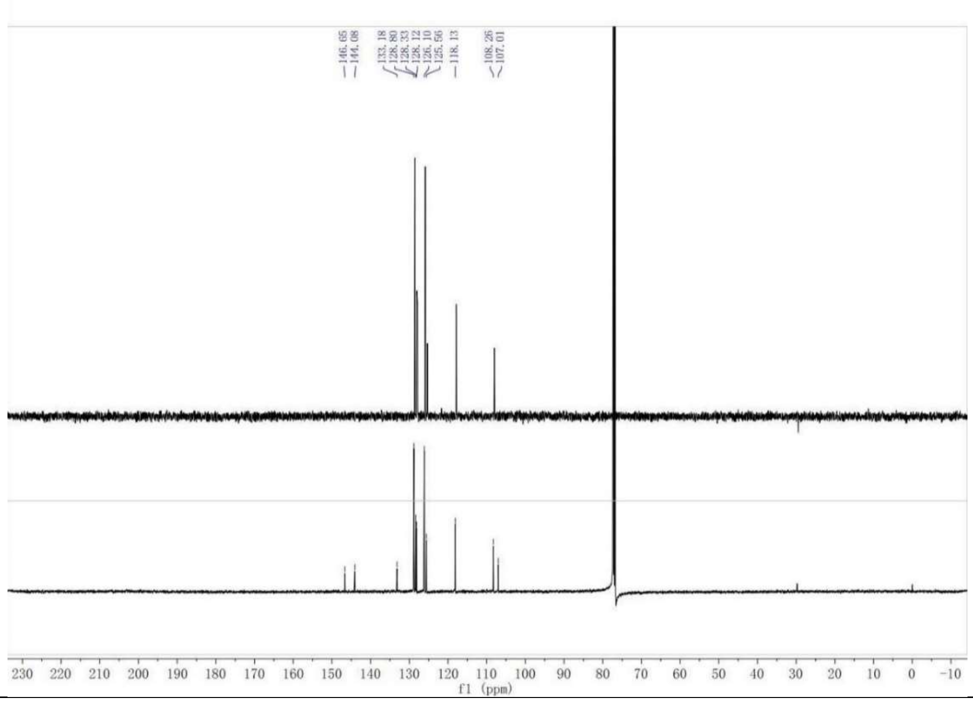

6-Bromo-2-phenylimidazo[1,2-a]pyridine (3s)

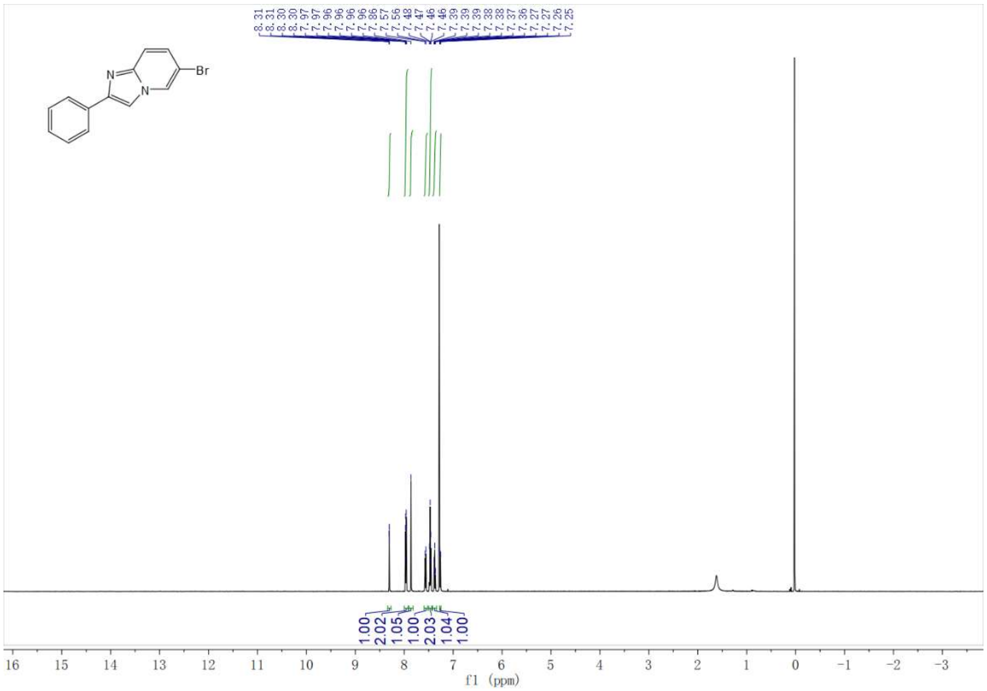

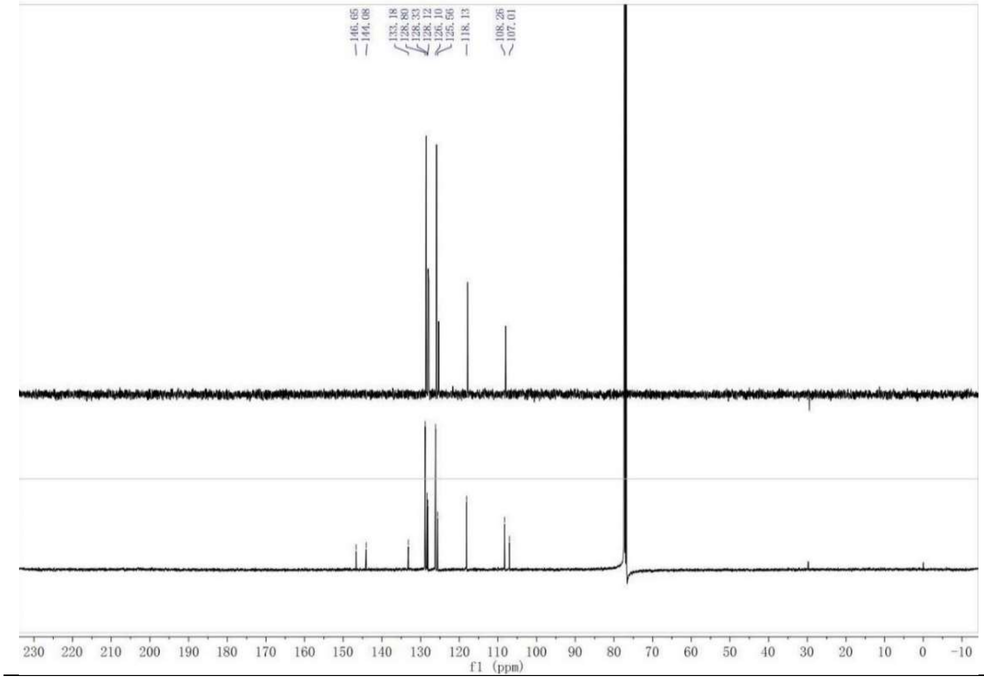

2-Phenyl-6-(trifluoromethyl)imidazo[1,2-a]pyridine (3t)

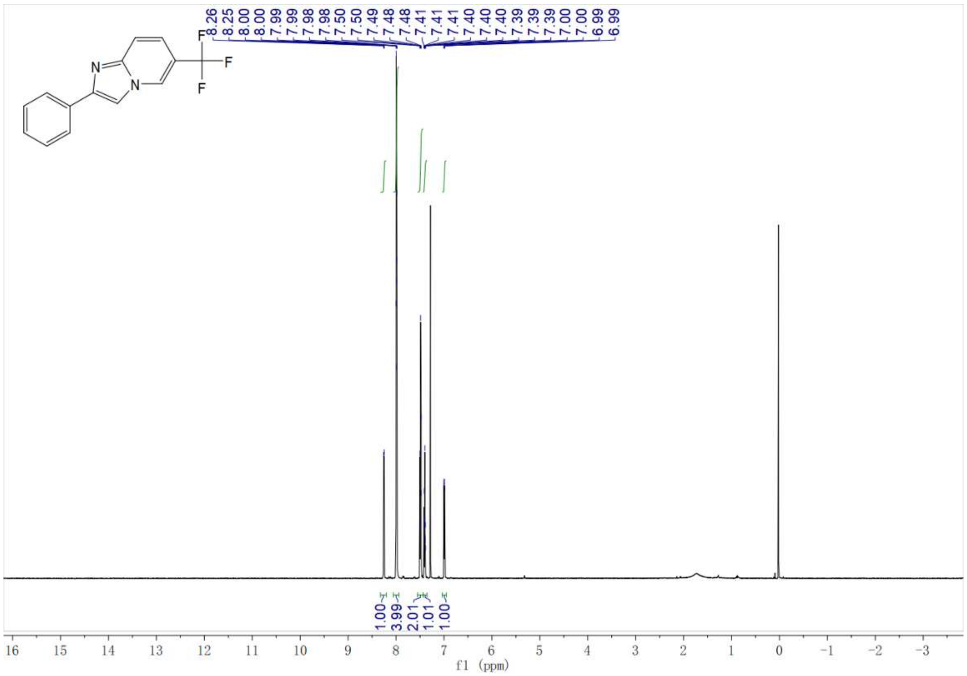

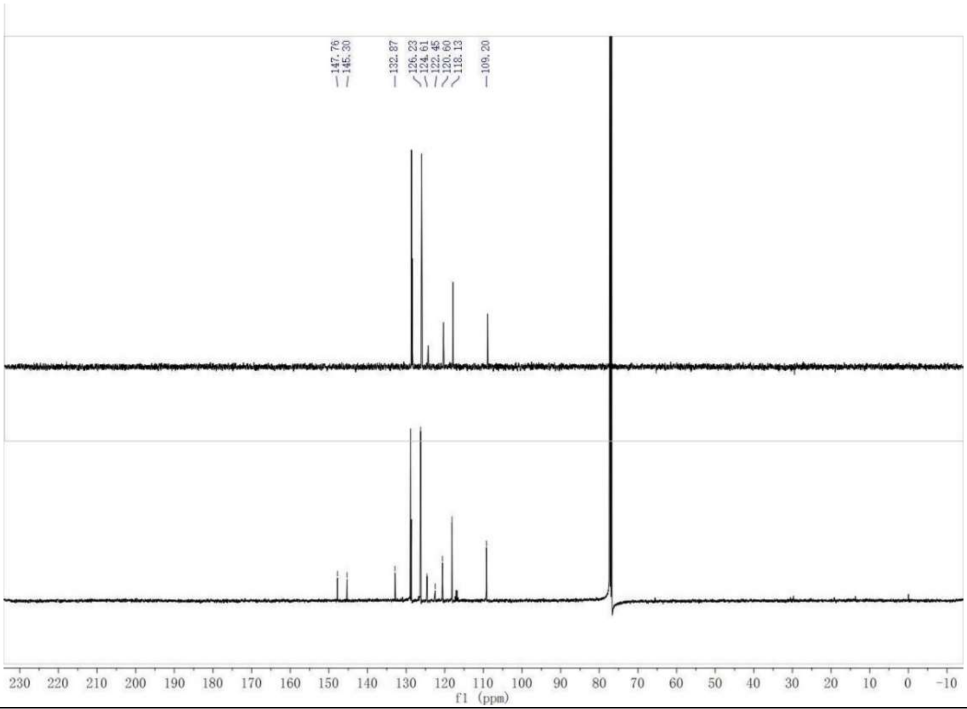

2-Phenylimidazo[1,2-a]pyridine-6-carbonitrile (3u)

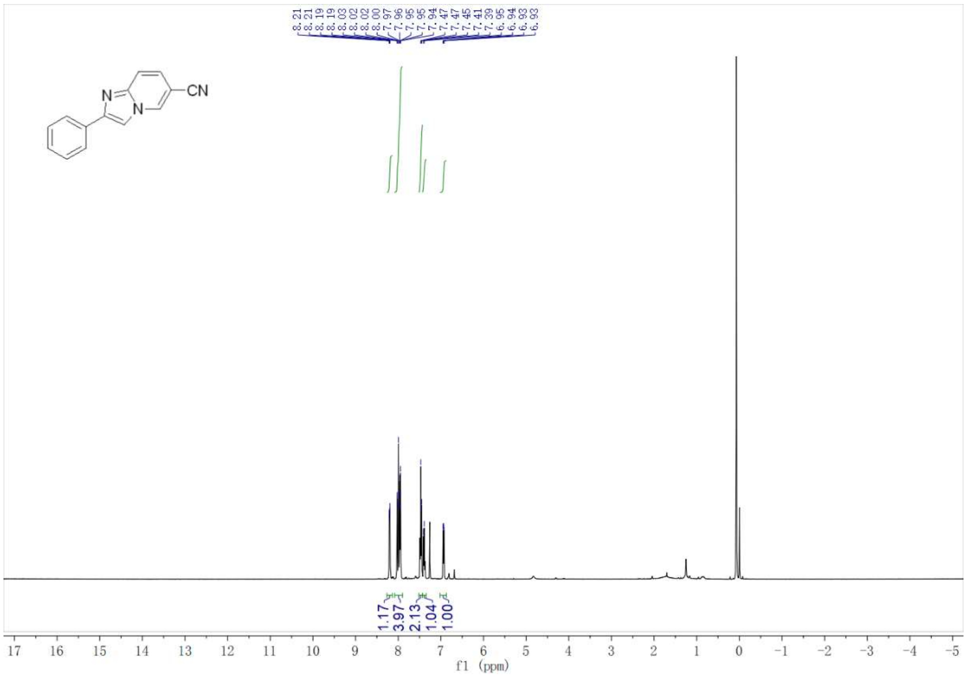

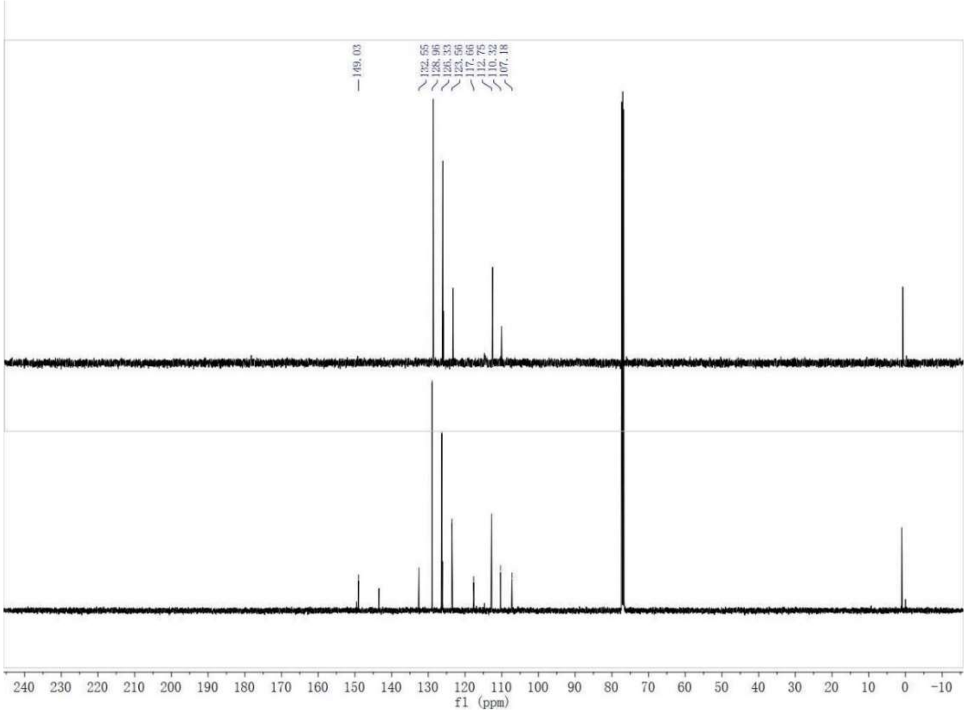

2-Phenylbenzo[d]imidazo[2,1-b]thiazole (5a)

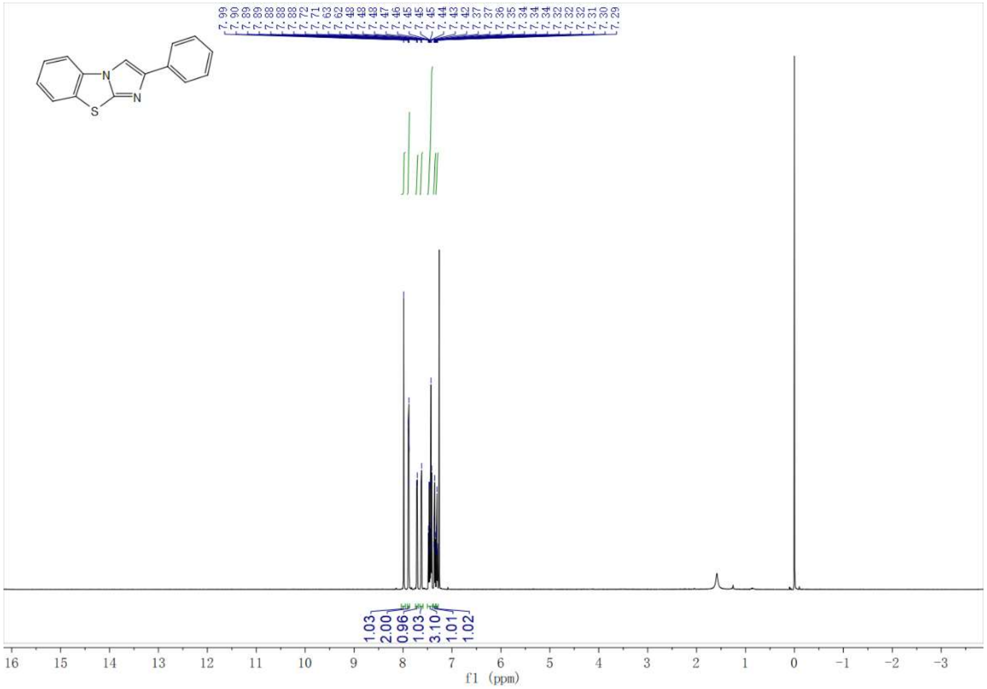

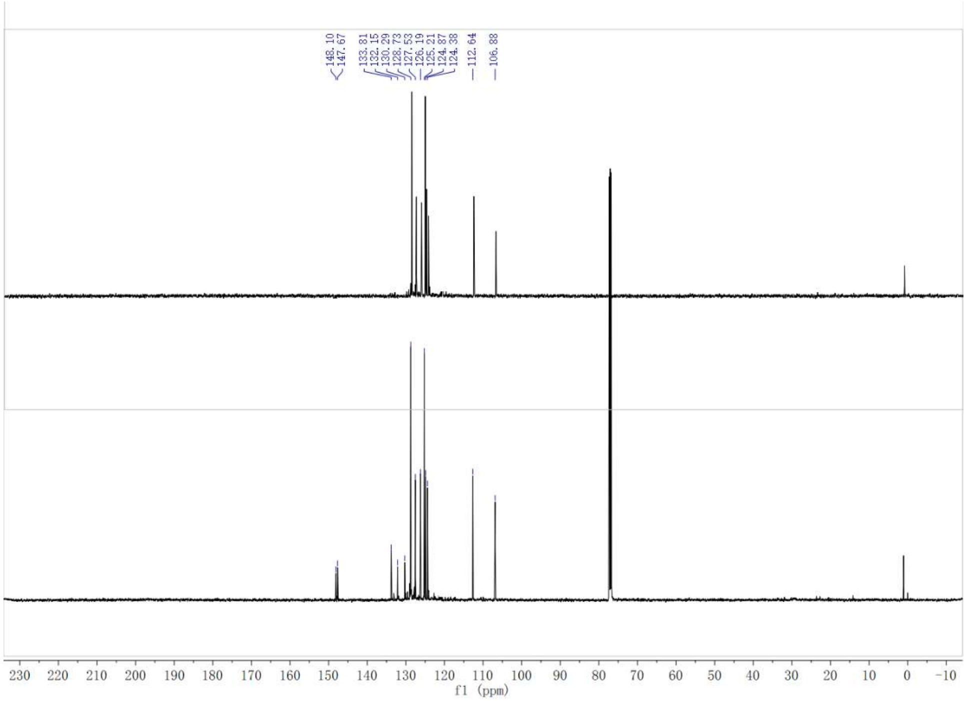

6-Phenylimidazo[2,1-b]thiazole (5b)

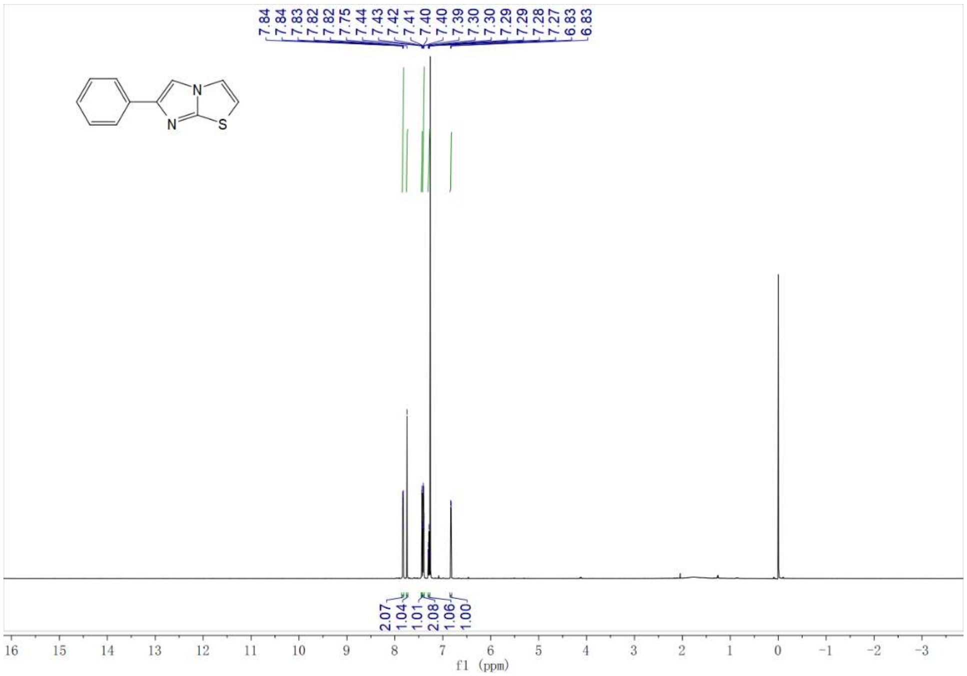

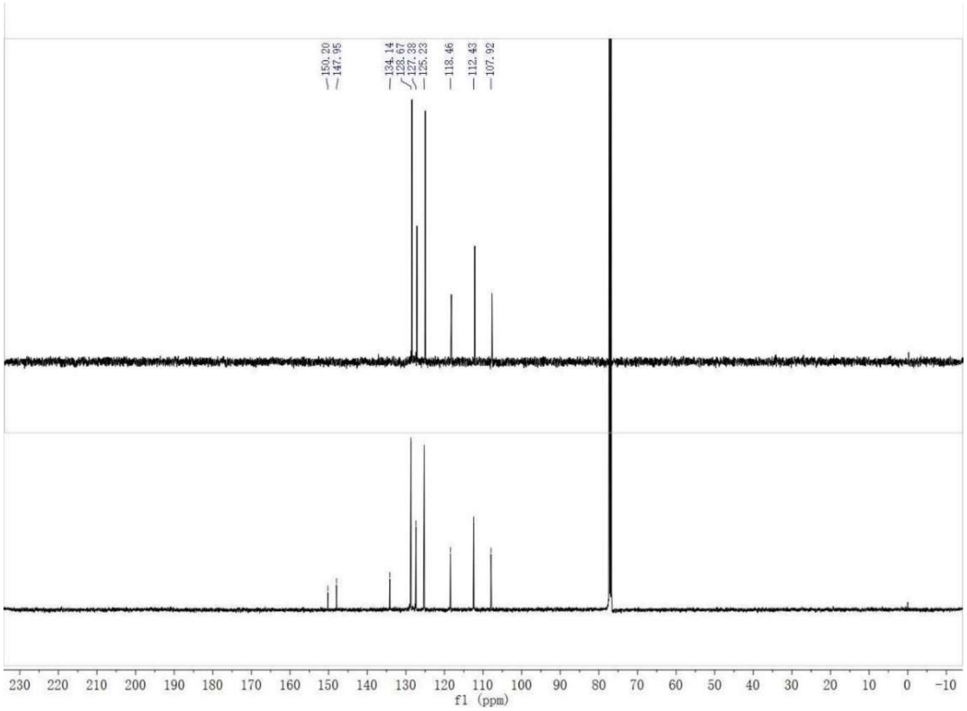

N,4-Diphenylthiazol-2-amine (5c)

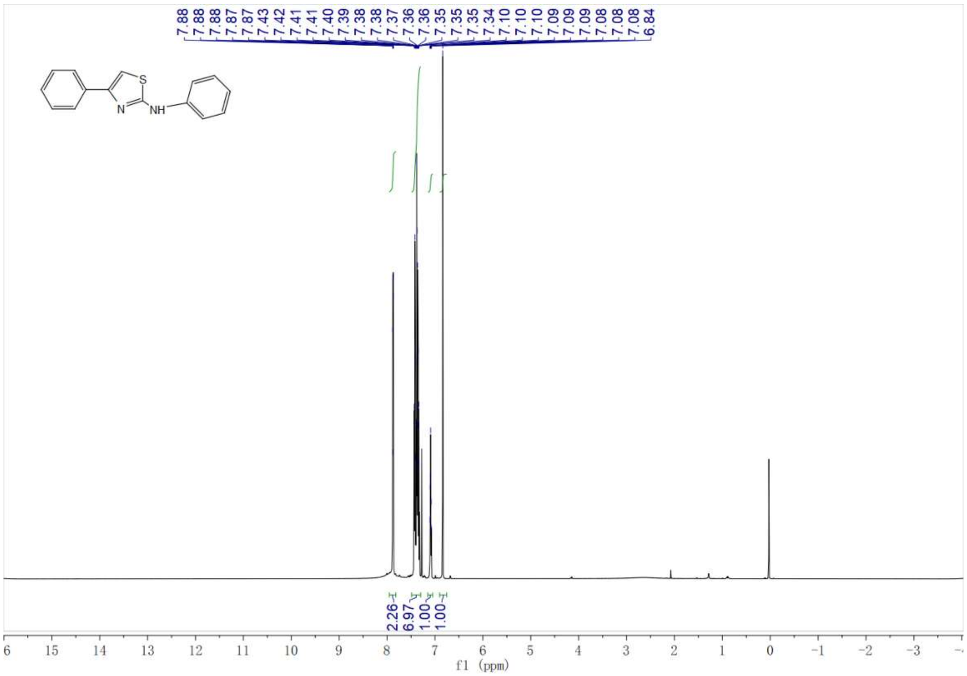

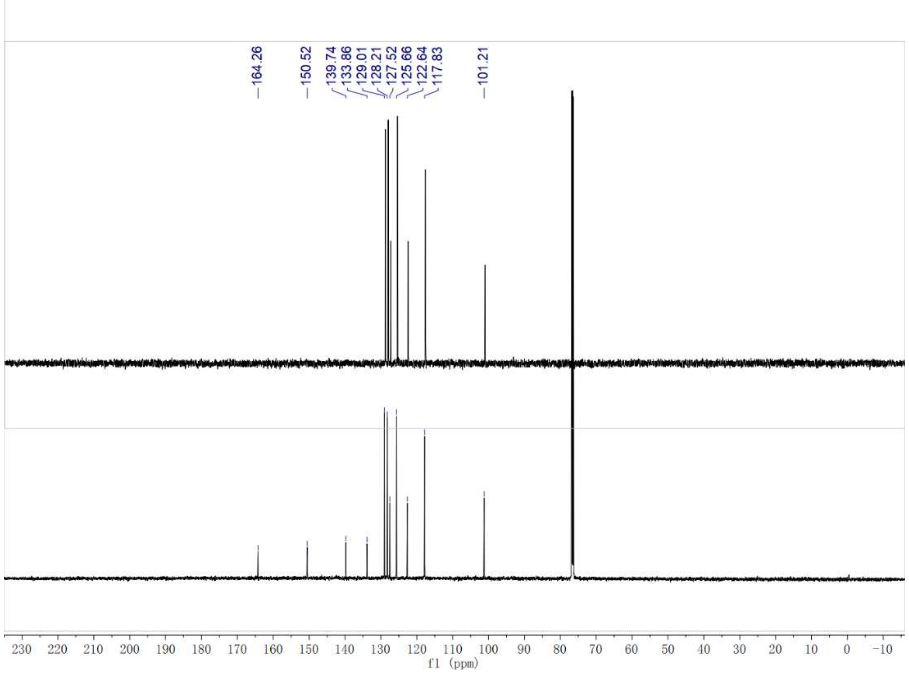

4-Phenylthiazol-2-amine (5d)

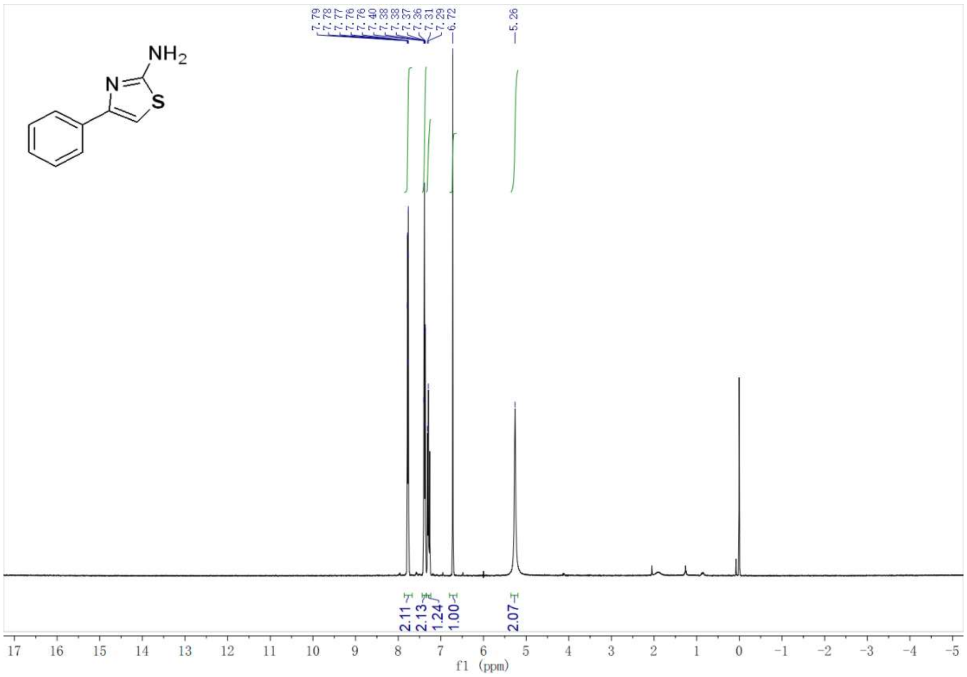

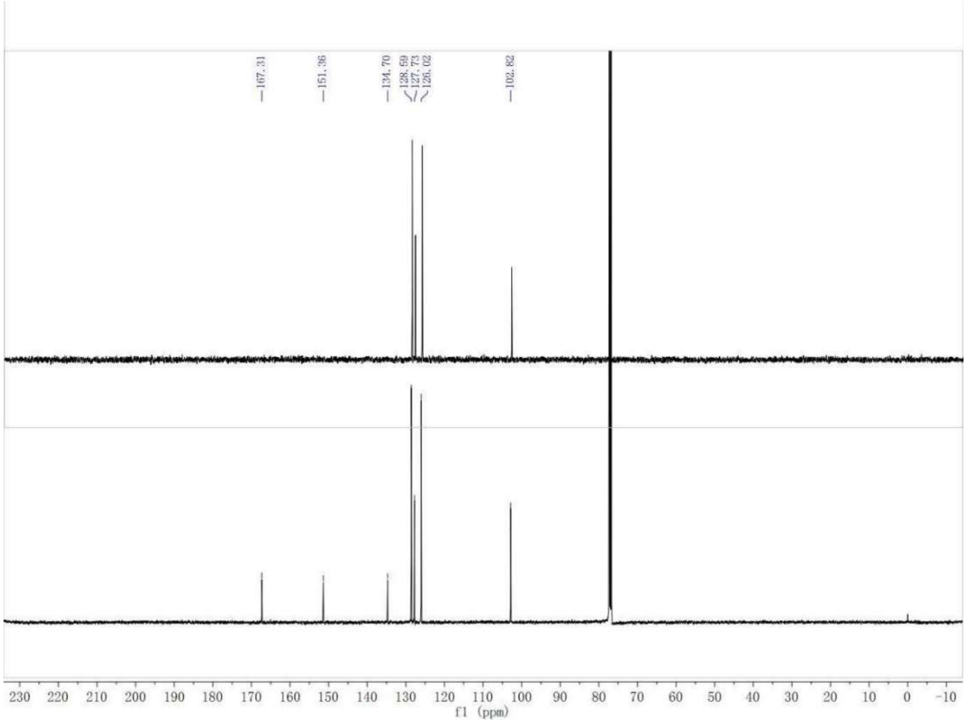

2-Methyl-4-phenylthiazole (5e)

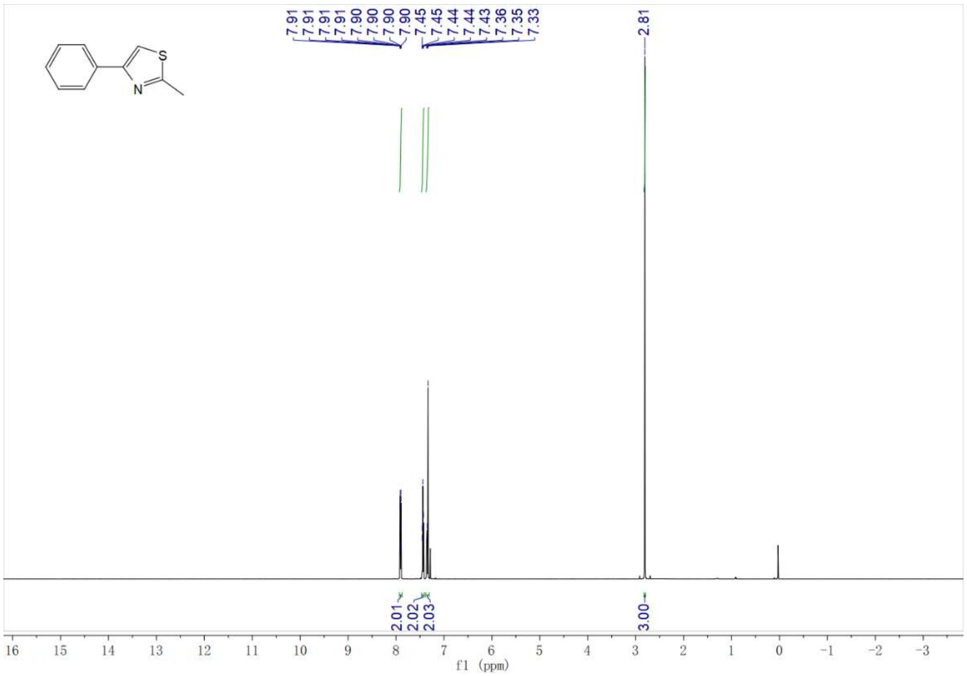

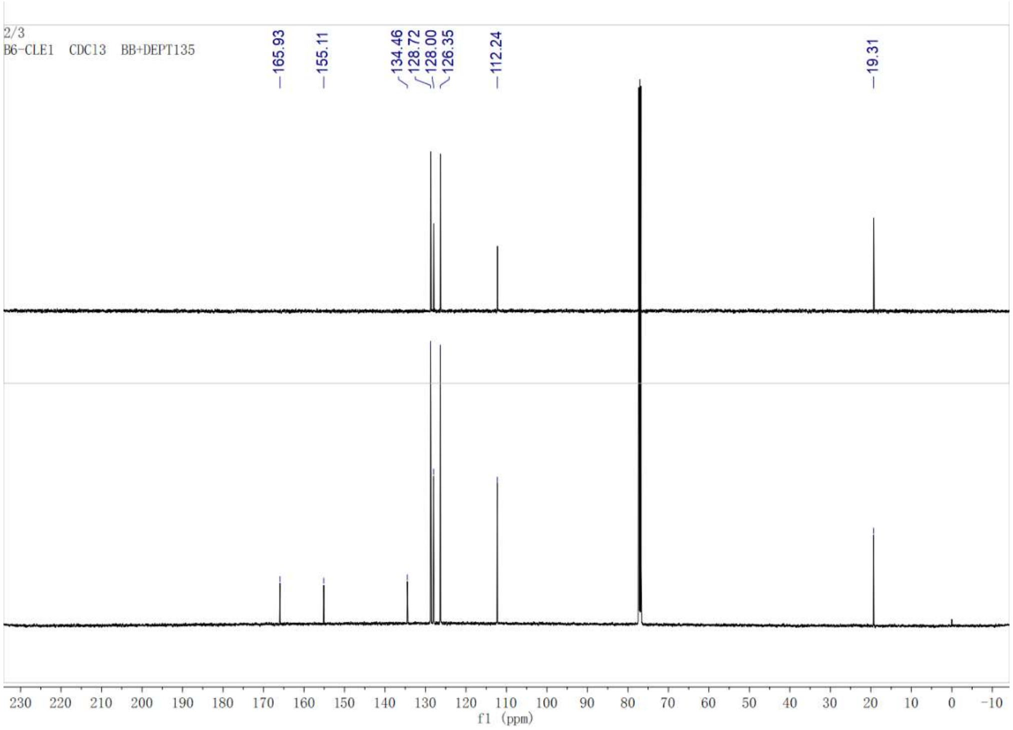

2,4-Diphenylthiazole (5f)

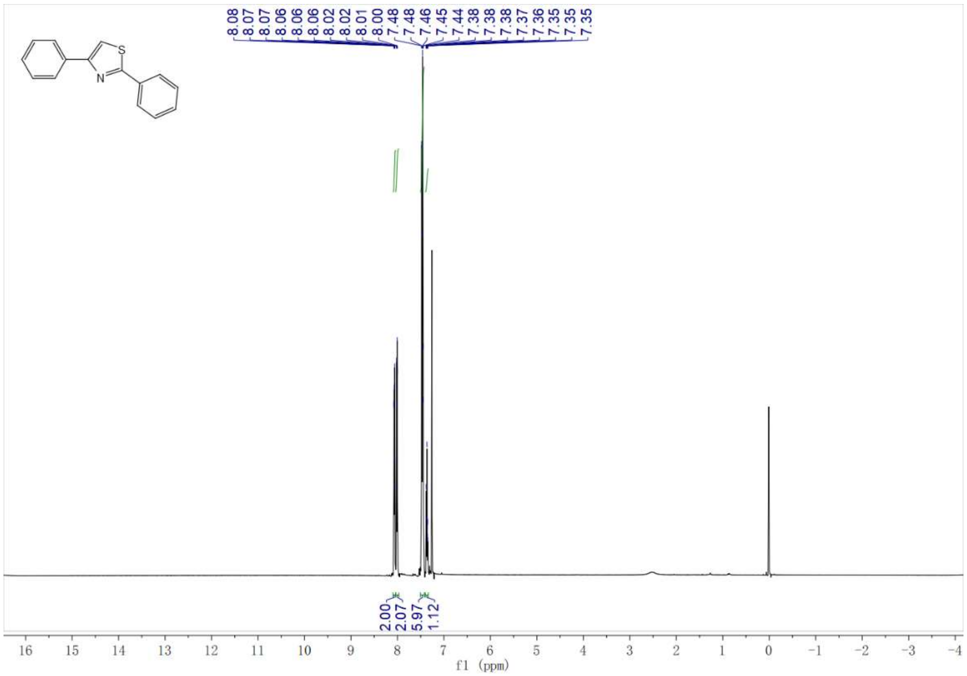

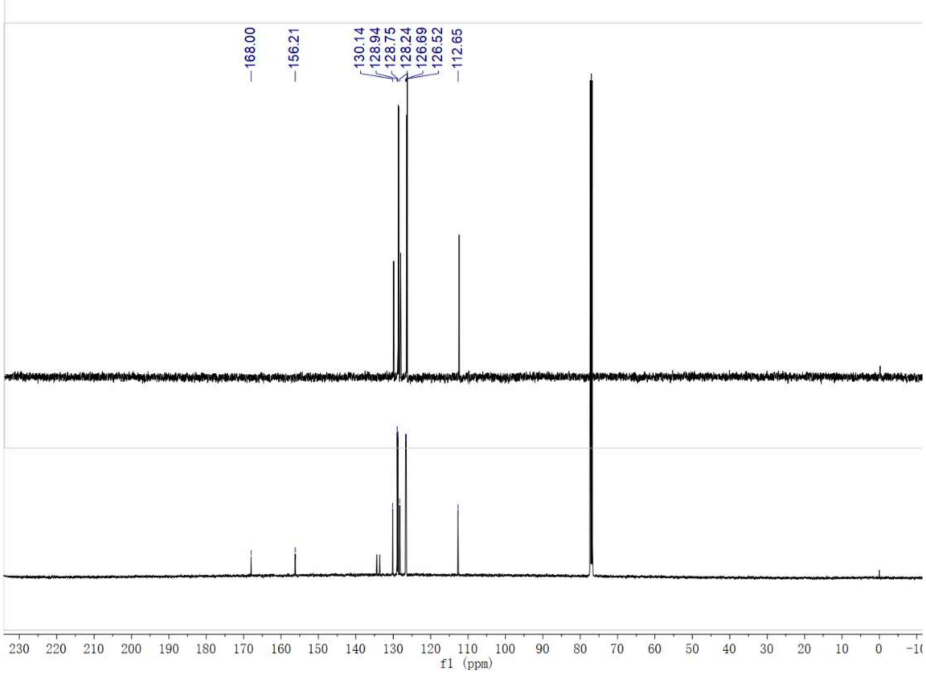

2-Phenylimidazo[2,1-a]isoquinoline (5g)

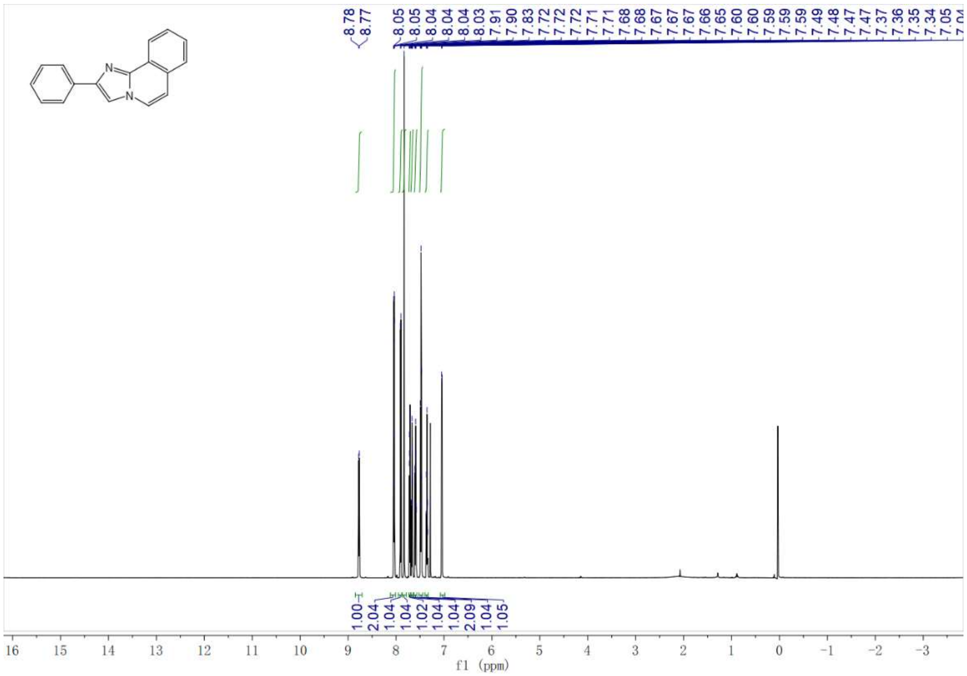

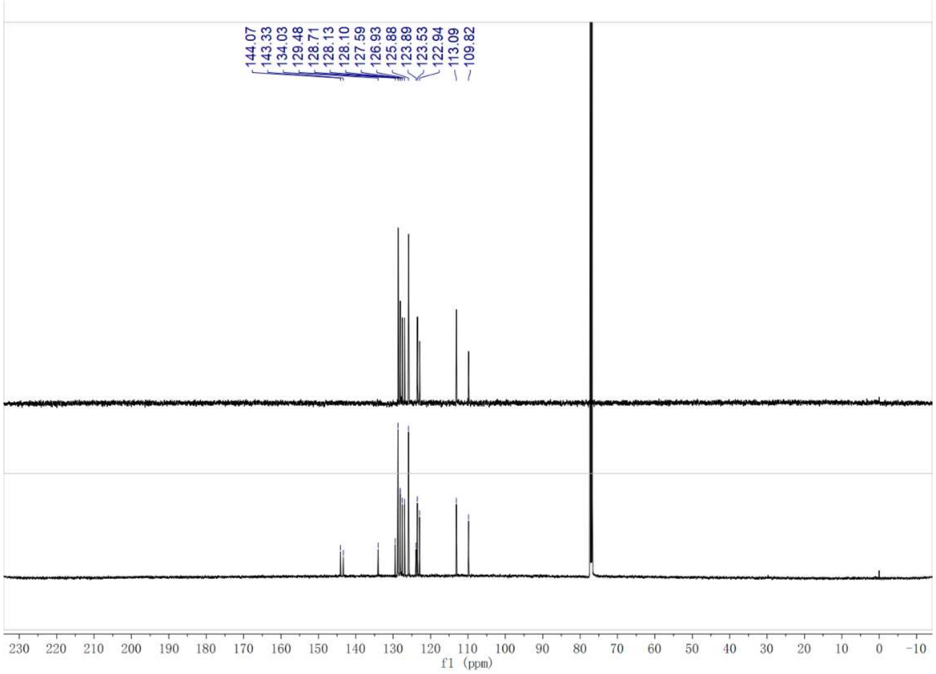

Supplement: Supplementary file 1 [file molecules-24-00893-s001.pdf]
